# Green synthesis of silver nanoparticles using *Carica papaya* L. flower extract for catalytic reduction of rhodamine B and biological activities

**DOI:** 10.1039/d6ra02406h

**Published:** 2026-07-15

**Authors:** Thi Tam Khieu, Thi Thao Truong, Thi Hong Tham Diep, Thi Thuy Ha Dinh, Ngoc Anh Hoang, Thanh Hai Cao, Tuan Kien Vu, Quang Tung Vu, The Chinh Pham, Le Phuong Hoang, Truong Xuan Vuong

**Affiliations:** a Faculty of Natural Sciences and Technology, TNU-University of Science Phan Dinh Phung Ward Thai Nguyen 25000 Vietnam xuanvt@tnus.edu.vn; b SGS Vietnam Co., Ltd Thai Nguyen Branch, Suoi Cat Hamlet, An Khanh Commune Thai Nguyen 2500 Vietnam; c Thai Nguyen University of Technology (TNUT) Tich Luong Ward Thai Nguyen 250000 Vietnam

## Abstract

A controlled and environmentally benign route for silver nanoparticle (AgNP) formation is achieved using an ethanolic–water (80 : 20) extract of male *Carica papaya* L. flowers as a combined reductant–stabilizer system. Reaction parameters, including pH, AgNO_3_ concentration, and extract dosage, are systematically varied to probe their influence on nucleation and growth behavior. Rather than treating phyto-synthesis as an end-point process, nanoparticle formation is examined as a time-resolved evolution governed by coupled nucleation and growth events. UV-vis spectroscopy reveals surface plasmon resonance bands at 405–425 nm, with spectral position and intensity strongly dependent on reaction conditions. Kinetic analysis using pseudo-first-order and Avrami models (*n* = 1–2) was consistent with discontinuous nucleation accompanied by surface-limited growth, with markedly accelerated formation under alkaline conditions. The Avrami-derived kinetic parameters further suggested a progression from predominantly nucleation-controlled behavior toward diffusion-influenced growth as the reaction evolved, providing a framework for interpreting structural development. X-ray diffraction confirms crystalline face-centered cubic Ag without detectable oxide phases. FTIR analysis identifies hydroxyl and carbonyl functionalities as key contributors to nanoparticle stabilization. TEM and XRD analyses reveal well-dispersed spherical particles with average sizes of 23.33 ± 6.44 nm and 26.21 ± 8.01 nm under optimized conditions. The resulting AgNPs mediate rapid NaBH_4_-assisted reduction of rhodamine B (97.7% removal of 20 mg L^−1^ within 60 min), following pseudo-first-order kinetics. Antibacterial activity is observed against *Escherichia coli*, *Staphylococcus aureus*, and *Pseudomonas aeruginosa*. Cytotoxic responses toward AGS and HepG2 cells (IC_50_ = 3.22 ± 0.57 and 2.05 ± 0.57 µg mL^−1^, respectively) were observed, indicating that surface-bound phytochemicals may remain functionally active after nanoparticle formation. Coupling kinetic descriptors with structural characteristics and functional performance supports a quantitative structure–kinetics–function relationship, suggesting that nanoparticle formation can be more systematically regulated through controlled synthesis variables rather than relying solely on empirical adjustment.

## Abstract 2

A controlled and environmentally benign route for silver nanoparticle (AgNP) formation is achieved using an ethanolic–water (80 : 20) extract of male *Carica papaya* L. flowers as a combined reductant–stabilizer system.

## Graphical abstract

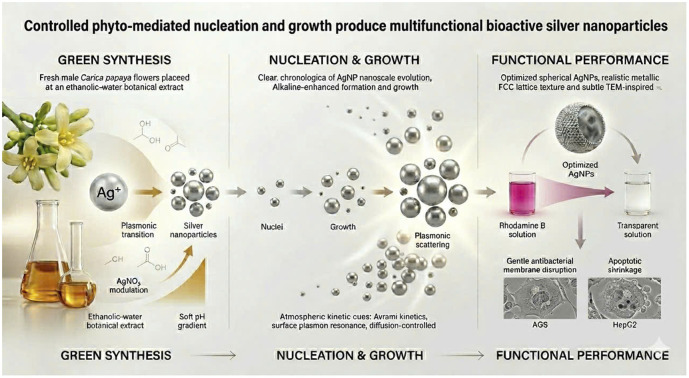

## 1 Introduction

Environmentally compatible synthesis strategies are increasingly reshaping nanoparticle fabrication in modern materials chemistry. Conventional preparation of metallic nanomaterials still relies heavily on strong chemical reductants, elevated temperatures, and synthetic stabilizers. Such conditions generate secondary waste streams and complicate implementation in systems requiring environmental safety and biological compatibility. Plant-mediated synthesis offers an alternative chemical environment in which naturally occurring metabolites participate directly in nanoscale assembly, functioning not only as electron donors but also as coordinating and stabilizing species during nucleation and particle maturation processes.^1–3^

Among metallic nanomaterials, silver nanoparticles (AgNPs) remain extensively investigated because of their optical response, catalytic activity, and antimicrobial properties. Their applications extend across water treatment, sensing technologies, catalysis, and biomedicine.^4,5^ In contrast to synthesis routes employing reductants such as hydrazine or sodium borohydride, phyto-mediated systems operate through chemically diverse phytochemical networks. Polyphenols, flavonoids, terpenoids, alkaloids, and organic acids interact simultaneously with silver precursors, generating coupled reduction–stabilization pathways that govern particle size, morphology, surface chemistry, and colloidal behavior.^1,2,6^

Numerous plant systems have been explored for AgNP synthesis. *Trigonella foenum-graecum* extracts generate AgNPs with catalytic and sensing functionality,^7^ whereas *Clerodendrum infortunatum* extracts facilitate rapid dye degradation processes.^8^ Similar observations have been widely reported throughout the phyto-mediated synthesis literature. However, most studies remain confined to endpoint characterization. Parameters such as particle size, antibacterial efficiency, or catalytic performance are commonly emphasized, whereas the kinetic sequence governing nanoparticle formation has received comparatively limited attention.^9–11^ Recent reviews have further noted that many phyto-mediated AgNP studies still lack quantitative mechanistic interpretation, reproducible structure–property relationships, and systematic kinetic analysis, thereby limiting predictive process control and scalable implementation.^4,12,13^ As a result, the formation pathway itself is still poorly resolved.

A comparable limitation exists in biomedical investigations. AgNPs synthesized using *Pistacia atlantica* and related medicinal plants exhibit antioxidant activity and cytotoxic responses toward cancer cell lines.^14,15^ Although such studies demonstrate promising biological functionality, the relationship between phytochemical composition and nanoparticle development is often insufficiently characterized. Consequently, empirical optimization continues to dominate much of the field.

Reaction environments strongly influence the structural development of phyto-mediated nanoparticles. Variations in pH, precursor concentration, extract composition, and extract dosage affect supersaturation behavior, nucleation frequency, and particle growth pathways, ultimately influencing morphology, colloidal stability, and structural uniformity.^2,16^ These effects become particularly difficult to predict in phytochemical systems characterized by extraction-dependent compositional heterogeneity. Without quantitative relationships linking synthesis parameters to nanoparticle evolution, reproducibility and rational process control remain challenging. Recent studies have identified phytochemical variability, insufficient synthesis standardization, and limited mechanistic predictability as major barriers preventing reliable scale-up of phyto-mediated nanoparticle production.^17,18^

Quantitative kinetic analysis provides an important route for understanding how nucleation and particle growth evolve during nanoparticle synthesis. Pseudo-first-order models describe overall reaction behavior, whereas the Finke–Watzky model separates nanoparticle formation into slow continuous nucleation followed by autocatalytic surface enlargement.^19^ However, under conditions where *k*_2_[Ag] ≫ *k*_1_, the model may produce inaccurate kinetic descriptions.^20^ The LaMer model describes burst nucleation followed by diffusion-controlled growth,^21^ although the heterogeneous and continuously evolving reaction environments characteristic of green synthesis systems rarely conform to this idealized behavior. Avrami analysis offers a complementary perspective by capturing nucleation characteristics, growth dimensionality, and structural progression during nanoparticle formation.^16^ Avrami-based approaches have previously been applied to thin-film AgNP systems^22^ and biomolecule-assisted nanoparticle synthesis.^23^ Yet their application to phyto-mediated AgNP synthesis remains relatively limited, particularly in studies linking kinetic descriptors with structural evolution and functional behavior.^18^

Integrating kinetic interpretation into controlled phyto-synthesis changes how nanoparticle evolution can be viewed. AgNP formation can then be interpreted not simply as a reduction process, but as a regulated transformation governed by nucleation kinetics, interfacial interactions, and structural constraints. Unlike many previous phyto-mediated AgNP studies that primarily emphasize endpoint performance, the present work focuses on the formation pathway itself through quantitative kinetic analysis. In this context, Avrami analysis provides insight beyond apparent rate descriptions by distinguishing nucleation-controlled and diffusion-limited regimes while simultaneously tracking changes in dimensional evolution during nanoparticle growth.^16^ Establishing relationships among synthesis conditions, kinetic descriptors, structural characteristics, and functional responses may therefore contribute toward a more predictive framework for green nanoparticle synthesis. Such integrated kinetic-structural interpretation remains comparatively underdeveloped in phyto-mediated AgNP systems, where mechanistic analysis is often disconnected from colloidal evolution and functional evaluation.

*Carica papaya* L. represents a chemically diverse phytochemical system that has received relatively limited attention in kinetically resolved nanomaterial synthesis. Leaves, seeds, and peels contain phenolic and flavonoid compounds capable of reducing metal ions while stabilizing evolving nanostructures through hydroxyl and carbonyl functionalities.^24–26^ Male *Carica papaya* flowers (MCF) have received substantially less attention despite longstanding applications in traditional medicine for hypoglycemic activity, gastric protection, and respiratory treatment.^27–29^ Cytotoxic responses toward cancer cell lines such as Michigan Cancer Foundation-7 (MCF-7) human breast cancer cells, HepG2 liver cancer cells, and KB epidermoid carcinoma cells have also been reported.^30,31^ These biological properties are associated with oxygen-containing metabolites capable of coordinating metal ions and participating in interfacial redox processes. Such metabolites may therefore influence not only biological activity, but also nanoparticle nucleation and structural development during synthesis. Existing studies on MCF remain largely confined to phytochemical screening and fragmentation analysis,^18^ while kinetically interpreted nanomaterial synthesis using this phytochemical system is not yet well established.

The coexistence of biological activity and nanoparticle-forming capability raises an important process-level question: can the metabolites responsible for medicinal activity also govern nucleation pathways, structural development, and functional responses during AgNP synthesis?

In this work, ethanolic extracts of MCF were employed as a combined reductant–stabilizer system for kinetically resolved AgNP synthesis. Phytochemical composition of the extract was examined alongside systematic variation of pH, precursor concentration, and extract dosage to evaluate their influence on nucleation and structural development. Formation kinetics were interpreted using pseudo-first-order and Avrami models to identify dominant growth regimes and nucleation characteristics. Structural evolution was characterized using UV-vis spectroscopy, XRD, FTIR, and SEM analyses, followed by evaluation of catalytic rhodamine B reduction, antibacterial activity, and cytotoxic responses. By integrating synthesis conditions, kinetic evolution, structural characteristics, and functional behavior within a single phyto-mediated nanoparticle system, this study aims to advance process-level understanding of green AgNP formation and support more predictive nanomaterial design strategies. This integrated approach contributes toward a more mechanistically informed framework for phyto-mediated AgNP synthesis.

## 2 Materials and methods

### 2.1 Chemicals

Silver nitrate (AgNO_3_, purity ≥99.8%), ethanol (C_2_H_5_OH, 96%, for HPLC), sodium carbonate (Na_2_CO_3_, purity ≥99.0%), sodium borohydride (NaBH_4_, purity ≥98%), RhB (C_28_H_31_N_2_O_3_Cl, purity ≥97%, for HPLC), *n*-butanol (*n*-C_4_H_9_OH, purity ≥99.5%), ethylene diamine tetra acetate disodium EDTA-2Na (C_10_H_14_N_2_Na_2_O_8_·2H_2_O, purity ≥99%), and AgNO_3_, (≥99.8%, Sigma-Aldrich) were purchased from Sigma-Aldrich, India, to be used without further purification. Double-distilled water (DW) was used throughout the study.

Male *Carica papaya* L. flowers were harvested in Thai Nguyen province in July.

### 2.2 Extracting male *Carica papaya* L. Flower and synthesising AgNPs

Male *Carica papaya* L. flowers (MCF) were collected in Thai Nguyen city, washed with distilled water, shade-dried, and ground into fine powder. MCF powder (10 g) was subjected to ultrasonic extraction with 80% ethanol at 60 °C for 60 min. The extract was centrifuged at 8000 rpm for 20 min at 5 °C and filtered through Whatman no. 1 filter paper. The filtrate was stored at 4 °C for further use and hereafter denoted as male *Carica papaya* flower extract (MCFE). Total polyphenols (TP) and total flavonoids (TF) of MCF were determined by the Folin–Ciocalteu method and the aluminium chloride (AlCl_3_) method.^32^

### 2.3 Green synthesis of AgNPs

MCFE was employed as both a reducing and stabilizing agent for the green synthesis of AgNPs. The reaction mixture consisted of 2.5 mL of MCFE and 1.5 mL of 0.025 M AgNO_3_ solution, which was diluted with deionized water to a final volume of 50 mL. The pH of the reaction mixture was adjusted to the desired value (7–10), and the solution was heated to 85 °C under continuous magnetic stirring.

The AgNO_3_ solution was added dropwise into the MCFE-containing solution to initiate the reduction of Ag^+^ ions and the formation of AgNPs. The reaction was maintained at 85 °C under constant stirring for a predetermined time. At specific time intervals, 3 mL aliquots were withdrawn from the reaction mixture and diluted to 10 mL with deionized water for UV-vis absorption measurements in the range of 300–700 nm using a UV-vis spectrophotometer (Jasco V-770).

After completion of the reaction, the suspension was cooled to room temperature and stored at 4 °C for further analysis. For structural characterization, a portion of the reaction mixture was centrifuged at 9000 rpm for 20 min at 5 °C. The obtained precipitate was collected, washed once with 96% ethanol and twice with deionized water to remove residual phytochemicals, and then dried. The dried product was finely ground and stored in a sealed container at room temperature for subsequent analyses.

The data of UV-vis spectroscopy were fitted with a Gaussian (340–600 nm) using peak analyzer and calculated *λ*_max_, peak area, with *R*^2^ ≫ 0.99, were subsequently fitted by the PFO and the Avrami kinetic models to describe the mechanism and rate of AgNPs formation. The PFO kinetic model postulates that the rate of AgNPs formation is directly proportional to the produced Ag content (corresponding to the peak area of the characteristic peak of Ag).^33^ The nonlinear form of the PFO equation is given as follows:

1 *A*_*t*_ = *A*_∞_(1 − e^−*k*_1_*t*^)

The Avrami kinetic model is commonly used in the crystallization processes exhibiting complex nucleation and growth behavior,^34^ given by:2
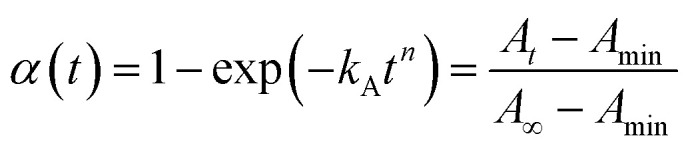


The linear equation of the Avrami model is:3
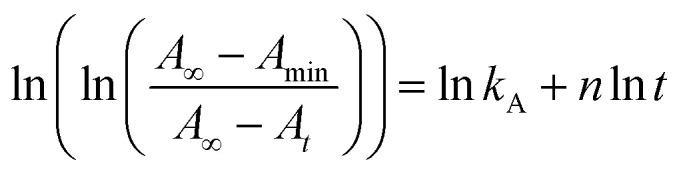
where *A*_*t*_, *A*_min_, *A*_∞_ are the peak area at time *t*, beginning at time zero (*t* = 0), and at equilibrium (*t* = ∞), respectively (*A*_∞_ is determined from the results of PFO model). *k*_1_ (min^−1^) and *k*_A_ (min^−*n*^) are the rate constants according to the PFO and Avrami kinetics. *n* is the Avrami exponent, which typically ranges from 1 to 4 and provides information regarding the nucleation/growth dimensionality. The kinetic parameters (*q*_e_, *k*_1_, *k*_A_, *n*, and *R*^2^) were assessed through a regression evaluation in OriginPro 2019.

### 2.4 Characterization of AgNPs

The crystalline structure of the synthesized silver nanoparticles (AgNPs) was analyzed using an X-ray diffractometer (XRD, Bruker AXS, Germany) equipped with Cu-Kα radiation (*λ* = 1.5406 Å) operated at 40 kV and 30 mA. Diffraction patterns were recorded over a 2*θ* range of 25–70°. The apparent crystallite size of AgNPs was estimated from X-ray diffraction (XRD) patterns using the Scherrer equation:

4 *D* = (*K* × *λ*)/(*β* × cos *θ*)

where *K* = 0.9 is the shape factor, *λ* = 0.15406 nm (Cu Kα radiation), *β* is the full width at half maximum (FWHM) of the diffraction peak (in radians), and *θ* is the Bragg angle.

The calculation was performed based on the diffraction peaks corresponding to the (111), (200), and (220) crystallographic planes of the face-centered cubic (fcc) structure of metallic silver. For measurement, the AgNPs suspensions were dropped onto glass slides, air-dried, and the coating process was repeated three times to ensure uniformity. The surface morphology of AgNPs was examined using a field-emission scanning electron microscope (FE-SEM, Hitachi S-4800, Japan) operated at an accelerating voltage of 20 kV. Before imaging, the samples were sputter-coated with a thin platinum layer for 30 s at 20 mA under a vacuum of 10 Pa to enhance conductivity. Functional groups associated with the surface of AgNPs were identified using Fourier transform infrared spectroscopy (FT-IR, IRTracer-100, Shimadzu, Japan) in the spectral range of 400–4000 cm^−1^ with a resolution of 1 cm^−1^. The samples were prepared as KBr pellets prior to measurement.

### 2.5 The catalytic reduction of rhodamine B (RhB)

The catalytic reduction of Rhodamine B (RhB) was performed with optimal AgNPs selected from the results of material characterization analysis (AgNPs7).

The first, 40 mL of RhB solution (20 mg L^−1^) was transferred into 100 mL Erlenmeyer flasks, followed by the addition of 3 mL of the prepared AgNPs7 suspension. Subsequently, varying volumes of 1 M NaBH_4_ solution (0.15, 0.30, 0.45, and 0.60 mL) were introduced, and the mixtures were shaken uniformly using a mechanical shaker under normal lighting conditions. At selected time intervals (10, 20, 30, 45, 60, and 90 min), 3 mL aliquots were withdrawn, diluted to 10 mL, and absorbance was measured at 553 nm using a UV-vis spectrophotometer with quartz cuvettes to evaluate the degradation efficiency of RhB.

Then, the optimum volume of NaBH_4_ solution was selected and used in the following experiments, following the same procedure, with initial RhB concentrations of 20, 30, 50, 75, or 100 mg L^−1^, and the amount of AgNP7 solution used being 1.5, 3.0, 4.5 and 6.0 mL. The RhB concentration in the reaction solution was determined at predetermined time intervals (5, 15, 30, 45, 60, and 90 min).

Similar experiments were performed under the condition of 40 m RhB 20 mg L^−1^ reacting with 3 mL AgNPs7 in the presence of 0.6 mL 1 M NaBH_4_ and some scavengers (0.1 M EDTA-2Na or *tert*-butanol (1 : 20) or 1 M AgNO_3_) to elucidate the electron-transfer-mediated catalytic reduction mechanism of RhB degradation by AgNPs.

The removal efficiency of RhB from the solution was calculated by the formula:5
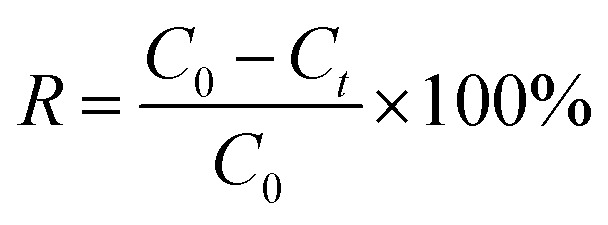
where *C*_0_, *C*_*t*_ (mg L^−1^) represent the RhB concentrations at *t* = 0 and *t* (minutes), *E* (%) denotes the efficiency of catalytic reduction.

Each experiment was performed in triplicate, and the results are presented as mean values. The catalytic reduction kinetics of RhB were evaluated using the Langmuir–Hinshelwood (LH), pseudo-first-order (PFO), and pseudo-second-order (PSO) kinetic models.^35,36^ The LH kinetic model is expressed as:6
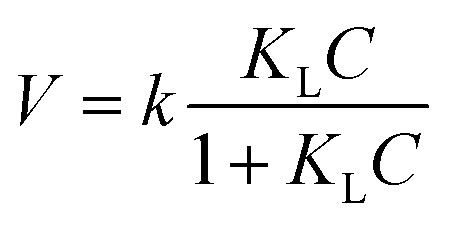


As *K*_L_*C* ≪ 1, the LH equation turns into the PFO kinetic model: *V* = *k*_1_*C*. The linear form of PFO kinetic models is followed by equations:7
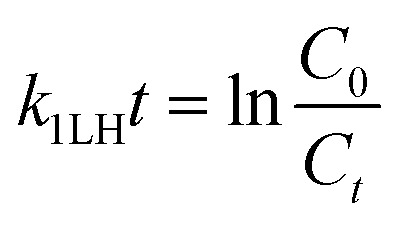


Additionally, the process kinetics can be described by PSO reaction kinetics according to the equation:^37^8
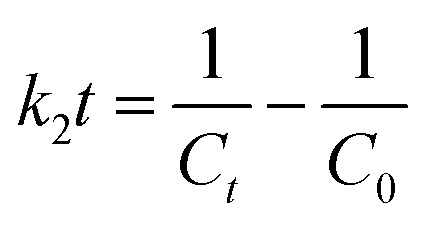
where *C*_0_ and *C*_*t*_ (mg L^−1^) are the RhB concentrations in the reaction solution at initial time and time *t* (min), *k*_1LH_ (min^−1^) and *k*_2_ (L mol^−1^ min^−1^) are the first and second order reaction rate constants respectively.

### 2.6 The antibacterial ability

AgNPs synthesized at pH 7 and pH 8, denoted as AgNPs7 and AgNPs8, respectively, were evaluated for antibacterial activity using the agar well diffusion method against Gram-positive *Staphylococcus aureus* ATCC 25923 and Gram-negative *Escherichia coli* ATCC 25922 and *Pseudomonas aeruginosa* ATCC 27853. The assays were performed using AgNP dispersions at 80 µg mL^−1^ in DMSO together with MCFE at 50 µg mL^−1^. Amoxicillin (100 µg mL^−1^) was used as the positive control.

Briefly, 100 µL of bacterial suspension (10^8^ CFU per mL) was uniformly spread onto Mueller–Hinton agar plates, followed by loading of 50 µL sample solutions into 6 mm wells formed in the agar medium. The plates were incubated at 37 °C for 24 h, after which the inhibition zone diameters were measured.

### 2.7 Cytotoxic activity on gastric cancer cell lines (AGS) and liver cancer cells (HepG2)

The AGS gastric cancer cell line and HepG2 hepatocellular carcinoma cell line used in this study were provided by Assoc. Prof. Dr Nguyen Phu Hung, Institute of Excellent Education and Research, Thai Nguyen University. These cell lines were originally obtained from the American Type Culture Collection (ATCC, Manassas, VA, USA).

80 µg mL^−1^ of AgNPs7 solution dispersed in water at 1000-, 100-, 40-, 20-, and 10-fold dilutions. They were tested on AGS and HepG2 cells by MTT assay. 100 µL of AGS or HepG2 cells suspension with concentration of 5 × 10^3^ cells per well was cultured for 48 h at 37 °C in a humidified atmosphere containing 5% CO_2_ and 95% humidified air in a 96-well microplate using RPMI 1640 medium supplemented with 10% fetal bovine serum (FBS) and 1% penicillin/streptomycin. After that, the cells were exposed to the test samples at different concentrations in the wells. Then, the plate was incubated for 48 h under the same conditions (37 °C, 5% CO_2_, and 95% humidified air) to evaluate the impact of the samples on cell proliferation. The cell viability was determined by the ability to reduce MTT to a formazan complex by the activity of mitochondrial dehydrogenase enzyme. The formazan product was dissolved in DMSO, and the optical density (abs) was measured at 570 nm, reflecting the rate of cell inhibition. Each concentration was tested in four replicate wells for each experiment. The cell proliferation rate was determined by the formula:9
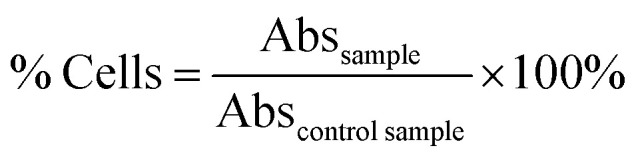


All experiments were performed in triplicate to ensure reproducibility.

## 3 Results and discussion

### 3.1 AgNPs characterization depending on synthesis process

#### 3.1.1 TP and TF of male *Carica papaya* L. flowers

The total polyphenol (TP) and total flavonoid (TF) contents of MCF, determined using the Folin–Ciocalteu and aluminium chloride (AlCl_3_) methods, were 24.006 mg GAE per g and 15.360 mg QE per g dry weight, respectively. Compared with previous reports, the TP content was higher than that reported for MCF collected in Madhya Pradesh, India,^38^ whereas the TF value was comparable to that reported for MCF from West Bengal, India.^39^

To further investigate phytochemical species potentially associated with nanoparticle formation, HPLC-DAD chromatographic analysis of the male *Carica papaya* flower extract was performed (Table S1 and Fig. S1 in SI). Based on retention-time correspondence with previously reported flavonoid profiles in *Carica papaya* extracts, the principal detectable chromatographic peaks were tentatively assigned to quercitrin, quercetin, and kaempferol. These hydroxyl-rich flavonoid species are chemically compatible with reductive and interfacial stabilization processes occurring during AgNP formation. Phenolic hydroxyl functionalities may participate in Ag^+^ reduction, while coordination interactions at the evolving nanoparticle interface could contribute to colloidal stabilization during nanoparticle growth. Within this framework, the detected flavonoid profile is consistent with the kinetically regulated nucleation–growth behavior inferred from the PFO and Avrami kinetic analyses. Comprehensive metabolite profiling together with quantitative determination of individual phytochemical constituents was not systematically performed in the present study and remains an important direction for future investigation.

#### 3.1.2 Optical evolution and kinetic analysis of AgNP formation

##### 3.1.2.1 Effect of pH and reaction time on AgNP formation

**Fig. 1:**
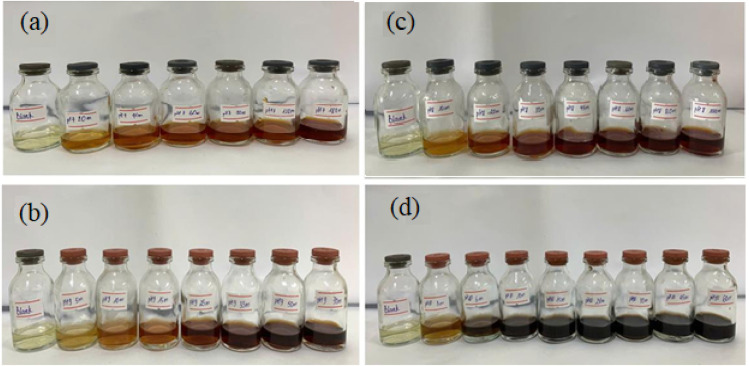
Color of AgNPs solution obtained over time at different pHs (a) pH 7, (b) pH 8, (c) pH 9, (d) pH 10.

**Fig. 2:**
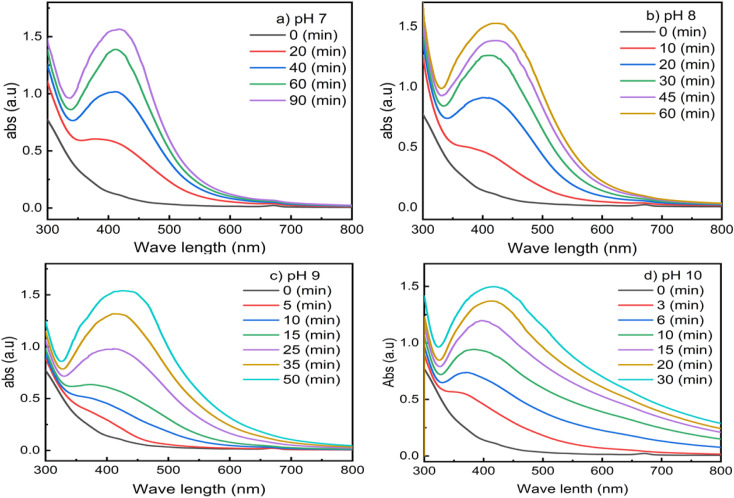
UV-vis spectra of silver nanoparticle (AgNP) synthesis over time at different pHs: (a) pH 7, (b) pH 8, (c) pH 9, (d) pH 10.

The reaction between 2.5 mL of MCFE and 1.5 mL of 0.025 M AgNO_3_ in 50 mL solutions was carried out at 85 °C at pH 7, 8, 9, 10 over time. The formation of AgNPs exhibited a strong dependence on pH and reaction time, as evidenced by the progressive color transformation and the emergence of localized surface plasmon resonance (LSPR) bands (Fig. 1 and 2).

The color change of the reaction mixtures demonstrates that AgNPs formation was strongly dependent on both reaction time and pH. At *t* = 0, all samples showed the pale-yellow color of the extract, indicating negligible Ag^+^ reduction. As time progressed, the solutions transitioned from pale-yellow to reddish-brown in deeper hues, consistent with the formation of nanoparticles and the onset of localized surface plasmon resonance (LSPR). The rate of color change increased substantially with increases in pH. The color change was rather slow at pH 7 and even at 180 min, the solution was still light brown. The brown color appeared quickly at pH 8–9, and at pH 10, a similar intensity of color developed within only 6 min, which was the equivalent of 25 min at pH 9 or 180 min at pH 7. These observations suggest that alkaline conditions accelerate Ag^+^ reduction and nanoparticle evolution within the colloidal system.

The UV-vis spectra of the reaction solutions as a function of pH and time are shown in Fig. 2.

At the initial stage (*t* = 0), the absence of a characteristic plasmon band (350–450 nm) confirmed a negligible reduction of Ag^+^. With increasing reaction time, a distinct LSPR band gradually appeared and intensified, indicating nucleation followed by nanoparticle growth.

**Fig. 3:**
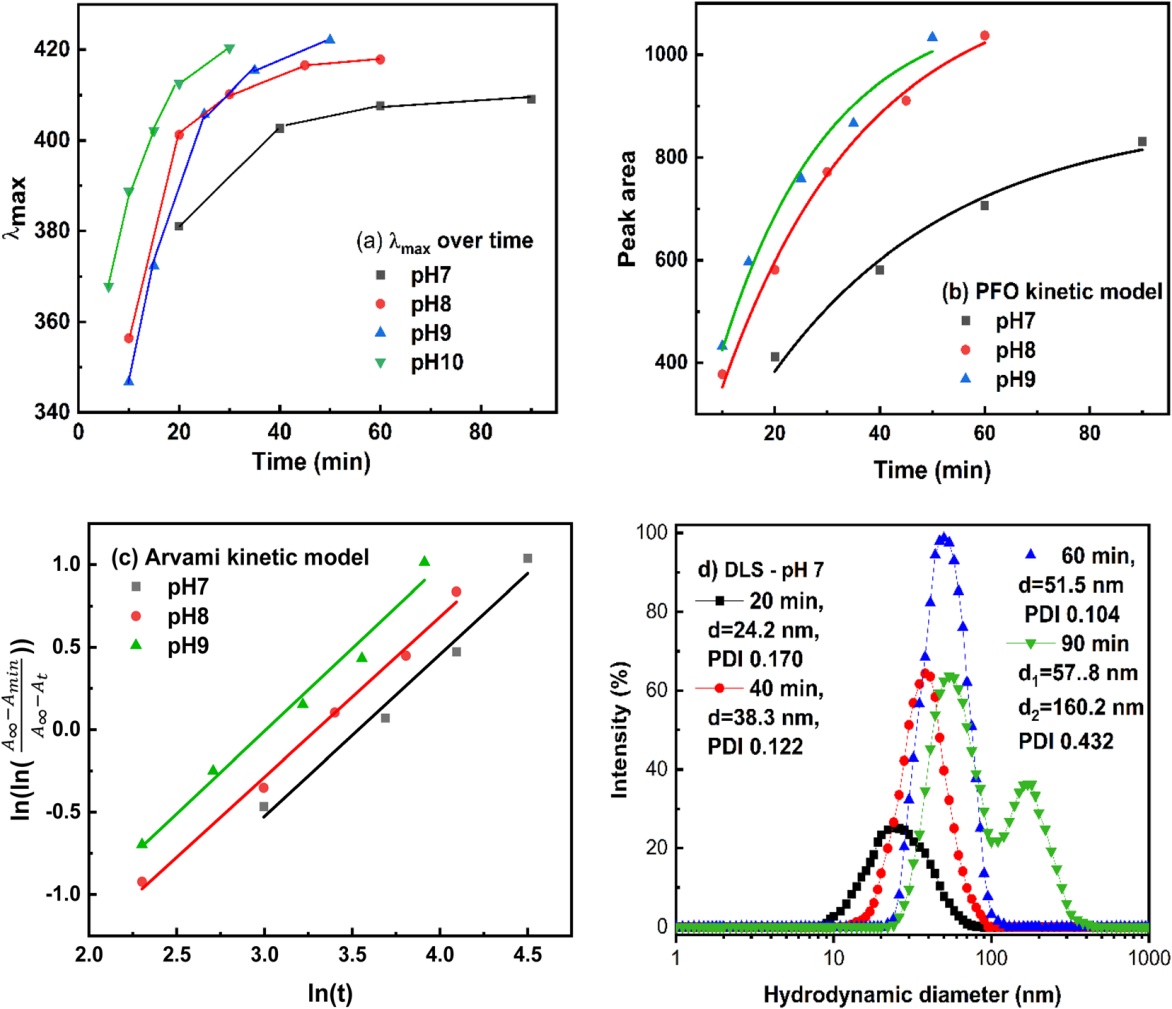
The shift of *λ*_max_ with pH and reaction time (a); the change of Ag peak area over pH and reaction time and fitting to PFO (b) and Arvami (c) kinetic models and (d) dynamic light scattering (DLS) particle-size distributions at different reaction times.

More importantly, the systematic red-shift of *λ*_max_ with increasing pH (Fig. 3a) provides direct insight into particle size evolution. In plasmonic systems, a red-shift is typically associated with an increase in particle size, increased interparticle coupling and broader size distribution. Thus, the accelerated red-shift observed at pH 9–10 indicates faster nucleation accompanied by rapid growth and partial aggregation. Our observations agreed with previous studies reporting pH-accelerated reduction and growth of AgNPs.^40–42^

Simultaneously, an increase in pH leads to faster spectral broadening and a more rapid loss of peak sharpness. The broadening of the LSPR band (increased FWHM) reflects growing polydispersity. In controlled nanoparticle synthesis, a narrow and symmetric peak indicates relatively narrow particle-size distribution, whereas broadening implies heterogeneous nucleation and secondary growth events. The pronounced spectral broadening under alkaline conditions therefore, indicates that while reduction kinetics are enhanced, size control becomes compromised. This observation is consistent with the color change of the solution shown in Fig. 1. Despite the relatively low peak intensity, the darker coloration indicates an increase in particle size. At pH 10, the increase in absorption is particularly rapid, with the LSPR band becoming intense within minutes and also at wavelengths greater than 500 nm. This behavior can be mechanistically explained by deprotonation of phenolic and carboxyl groups in MCFE under alkaline conditions, increasing their electron-donating capability and accelerating Ag^+^ reduction. However, the rapid generation of nuclei likely exceeds the stabilization capacity of phytochemicals, resulting in partial coalescence.

Overall, the UV-vis evolution may reflect that alkaline conditions accelerate Ag^+^ reduction and nanoparticle growth, while simultaneously promoting interparticle interactions and partial aggregation at prolonged reaction times. These spectroscopic observations were further correlated with kinetic fitting analysis to better resolve the nucleation–growth behavior of the phyto-mediated AgNP system.

##### 3.1.2.2 Kinetics of AgNPs formation

To further elucidate the formation mechanism, peak area evolution was analyzed using pseudo-first-order (PFO) and Avrami kinetic models (Fig. 3b, c and Table 1), according to the following eqn (1) and (3).

**Table 1:** Kinetic parameters of green synthesis AgNPs using MCFE

pH	PFO			Avrami		
	*A*_∞_	*k*_1_	*R* ^2^	*n*	*k*_A_	*R* ^2^
7	883.71	0.0285	0.982	1.65	0.031	0.997
8	1151.46	0.0366	0.995	1.50	0.046	0.995
9	1102.98	0.0486	0.986	1.35	0.081	0.996

The *R*^2^ values from Table 1 and Fig. 3b, c demonstrated satisfactory agreement between the experimental data and both the PFO and Avrami kinetic models. PFO rate constants (*k*_1_ > 10^−2^ min^−1^) increased progressively with pH, reflecting faster Ag^+^ reduction in alkaline media. PFO fitting captures the global reduction kinetics, whereas the Avrami model offers a more detailed view of the nucleation–growth evolution.

Avrami exponents in the range of *n* = 1.35–1.65 are commonly linked to discontinuous nucleation processes influenced by interfacial effects and constrained growth behavior rather than unrestricted volumetric growth.^43^ In the MCFE-mediated system, phytochemical species may interact with the evolving AgNP surface during nucleation, restricting unrestricted isotropic growth and suppressing excessive particle coarsening under optimized conditions. A similar trend appears in the optical response. At pH 7, the LSPR band evolved gradually and remained relatively narrow, consistent with controlled nanoparticle growth and improved colloidal uniformity.

Reaction behavior changed substantially under strongly alkaline conditions (pH ≥ 9). Faster reduction promoted rapid supersaturation and high nucleation density. Particle–particle collision also became increasingly frequent. Although the Avrami-derived *n* values remained within the surface-influenced regime, spectral broadening, baseline elevation, and progressive red-shifting of the LSPR band pointed toward aggregation and diffusion-influenced interparticle interactions during later synthesis stages. Such behavior may reflect a gradual transition from predominantly surface-limited growth toward partially diffusion-mediated coalescence under highly alkaline conditions. The Avrami curve at pH 7 retained a gradual sigmoidal profile, while at pH 9 the transformation rapidly approached completion (*α* → 1) at early reaction times.^44^ Decreasing *n* values at elevated pH further imply that alkaline conditions alter not only the reduction rate but also the dominant growth pathway.

Neutral conditions (pH 7) produced a slower yet more stable growth regime. *λ*_max_ remained comparatively stable throughout the reaction period, and the FWHM stayed narrower over time, suggesting sustained colloidal stabilization. Around 60 min, peak-intensity growth slowed and the spectral baseline began to rise. Deviation from idealized Avrami behavior beyond this point likely originated from interparticle interaction and aggregation processes that are not fully described by the classical Avrami framework. For this reason, pH 7 and 60 min were selected as the most balanced conditions for simultaneous rate control and particle-size regulation.

DLS measurements further supported the kinetic interpretation (Fig. 3d). The hydrodynamic diameter increased progressively with reaction time while maintaining low PDI values (≤0.170) up to 60 min, consistent with controlled nanoparticle growth and sustained colloidal stability. At prolonged reaction times (90 min), the emergence of a second particle population together with a marked increase in PDI indicated increasing aggregation, in agreement with the concurrent UV-vis spectral broadening. A more detailed discussion of colloidal evolution is presented in Section 3.1.3.

Kinetic fitting, spectroscopic evolution, and DLS analysis collectively support the view that MCFE contributes not only to Ag^+^ reduction but also to colloidal stabilization and growth regulation during AgNP formation. Among the investigated conditions, pH 7 provided the most stable balance between reduction kinetics, colloidal stability, and particle-size control. The present catalytic analysis nevertheless remains primarily comparative rather than fully quantitative, since catalyst-normalized apparent rate constants and long-term catalyst stability were not systematically evaluated in the current colloidal system.

#### 3.1.3 DLS analysis and colloidal evolution of AgNPs

To further clarify the colloidal evolution of the biosynthesized AgNPs, dynamic light scattering (DLS) analysis was performed on the AgNP suspension synthesized at pH 7 at different reaction times (Fig. 3d). AgNPs collected at 20, 40, and 60 min exhibited average hydrodynamic diameters of 24.2, 38.3, and 51.5 nm, respectively, with corresponding PDI values of 0.170, 0.122, and 0.104. According to ISO 22412, PDI values below 0.2 correspond to monodisperse colloidal systems, whereas higher values reflect increasing polydispersity. Progressive particle-size growth accompanied by relatively low PDI values points to controlled nanoparticle evolution during the early and intermediate synthesis stages. The same general tendency appeared in the UV-vis spectra through gradual LSPR red-shifting. However, although this spectral evolution is broadly consistent with nanoparticle growth, contributions from interparticle plasmon coupling and local colloidal interactions cannot be completely excluded. At 60 min, the colloidal system reached a hydrodynamic size of ∼51.5 nm together with a PDI of 0.104, corresponding to a comparatively narrow particle-size distribution. This stage also closely matched the Avrami conversion approaching saturation (*α* → 1), consistent with a relatively stable nucleation–growth regime. By 90 min, two distinct particle populations (∼57.8 and ∼160.2 nm) emerged alongside a sharp increase in PDI to 0.432, indicating the onset of substantial colloidal heterogeneity. Under prolonged reaction conditions, aggregation and interparticle association likely became increasingly important contributors to the observed colloidal behavior. These features were consistent with the concurrent spectral broadening and plasmonic distortion observed in the UV-vis analysis. Larger aggregates may additionally enhance light scattering within the colloidal suspension, contributing to the darker brown coloration observed at extended reaction times. Nevertheless, because UV-vis spectroscopy and DLS primarily provide indirect ensemble-level information, complete differentiation between nanoparticle growth, interparticle coupling, and aggregation processes remains challenging without complementary high-resolution time-resolved imaging analyses.

#### 3.1.4 Effect of extract volume and Ag^+^ concentration on AgNPs formation

**Fig. 4:**
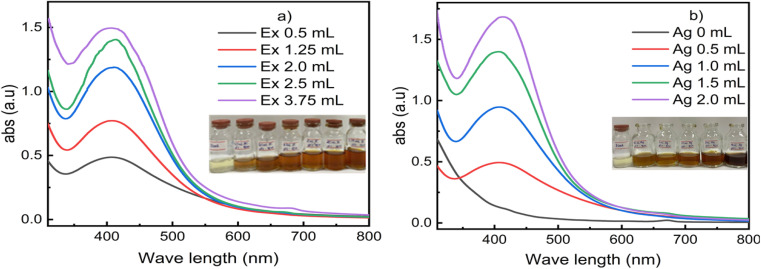
UV-vis spectrum and color of AgNPs synthesis reaction solution with different amount of extract (a) and amount of Ag salt (b).

The extract volume primarily modulated stabilization efficiency. Increasing MCFE volume (0.5–2.5 mL) enhanced plasmon intensity without significant *λ*_max_ shift (Fig. 4a), indicating increased particle number rather than size growth. The preservation of peak symmetry suggests efficient surface passivation.

The extract volume primarily modulated stabilization efficiency. Increasing MCFE volume (0.5–2.5 mL) enhanced plasmon intensity without significant *λ*_max_ shift (Fig. 4a), indicating increased particle number rather than size growth. The preservation of peak symmetry suggests efficient surface passivation. Beyond 2.5 mL, spectral broadening and solution darkening were observed, implying the onset of aggregation. Excess phytochemicals may induce interparticle bridging or alter ionic strength, reducing colloidal stability.

Conversely, increasing AgNO_3_ concentration (Fig. 4b) significantly influenced nucleation density. At moderate Ag^+^ levels (1.5 mL), a sharp and slightly blue-shifted plasmon band was obtained, suggesting formation of smaller and well-dispersed particles due to balanced nucleation and stabilization. At excessive Ag^+^ (2.0 mL), the red-shift and peak broadening indicate accelerated growth and aggregation. The spectrophotometric saturation further confirms high particle density and possible coalescence. These findings demonstrate that Ag^+^ availability governs nucleation density, while extract concentration controls surface stabilization. The interplay between these two parameters defines the final particle size distribution and colloidal stability. The optimum was 1.5 mL AgNO_3_, producing a sharp plasmon peak and stable colloid with small, uniform particles.

Collectively, the combined plasmonic evolution and kinetic modeling reveal that MCFE-mediated AgNP synthesis follows a pH-dependent discontinuous nucleation mechanism with surface-limited growth.

### 3.2 Characterization of AgNPs

**Fig. 5:**
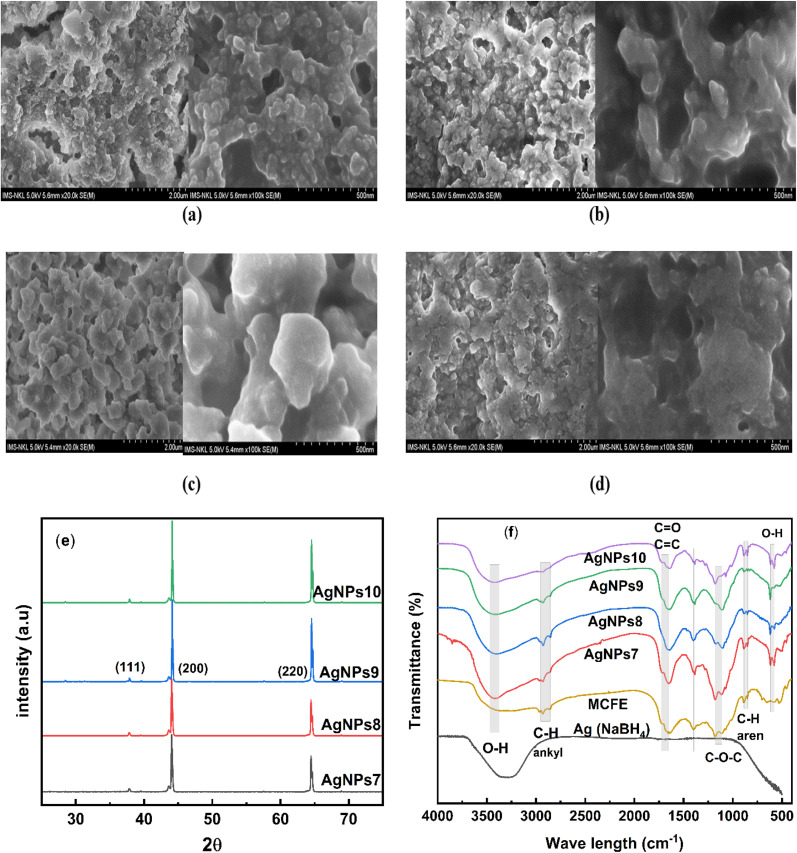
Some characteristics of AgNPs: SEM images of AgNPs samples formed at different pH: (a) pH 7 – AgNPs7, (b) pH 8 – AgNPs8, (c) pH 9 – AgNPs9 and (d) pH 10 – AgNPs10; (e) XRD spectra and (f) FTIR spectra.

The characteristics of AgNPs were analyzed by X-ray diffraction, FTIR and SEM techniques, shown in Fig. 5.

#### 3.2.1 Morphology and particle-size characteristics of AgNPs

##### 3.2.1.1 SEM analysis of AgNPs

SEM analysis provided further insight into the effect of pH on nanoparticle morphology and aggregation behavior (Fig. 5a–d). AgNPs synthesized at pH 7 (AgNPs7) exhibited predominantly spherical morphology with slight surface roughness, together with relatively uniform particle distribution and minimal aggregation. When the pH was raised to a value of 8, particles (AgNPs8) exhibited some aggregation as small clusters formed on the surface of otherwise smooth particles. Higher magnifications revealed that the particles had a relatively uniform size of ∼30–50 nm, which suggests their growth was controlled yet appeared less stable than AgNPs7. By pH 9, the particles (AgNPs9) began to exhibit significantly larger sizes, where initial continuous domains became aggregates of ∼300 nm. The morphology indicates the colloidal system became destabilized with stronger alkaline conditions as reduced electrostatic repulsion drove more particles to coalesce. The trend was more pronounced at pH 10, where the sample (AgNPs10) displayed a porous but highly heterogeneous structure. Although some primary particles appeared smaller than those in AgNPs9, they were fused into large, irregular agglomerates, making individual boundaries difficult to resolve. This observation correlates well with kinetic analysis, reinforcing the link between reaction rate, nucleation mechanism, and final morphology.

To gain deeper insight into the nanoscale structure and particle size distribution, transmission electron microscopy (TEM) was employed.

##### 3.2.1.2 TEM analysis of AgNPs

**Fig. 6:**
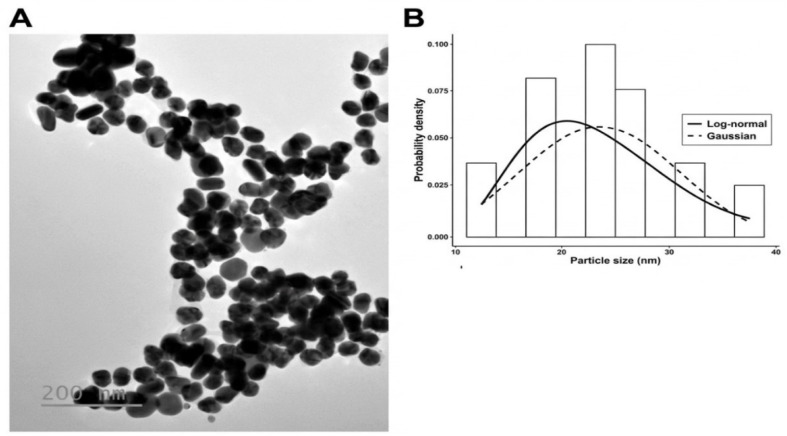
Representative Transmission Electron Microscopy (TEM) analysis of the synthesized nanoparticles (A) and particle size distribution of AgNPs with Gaussian and log-normal fitting (B).

To further evaluate particle-size distribution and colloidal evolution, statistical analysis of TEM-derived nanoparticle dimensions was performed using Gaussian and log-normal fitting models (Fig. 6B). The resulting histogram exhibited a slightly asymmetric distribution centered around ∼20–25 nm, indicating moderate polydispersity within the colloidal system.

The TEM micrographs (Fig. 6) reveal detailed morphological features, particle size, and spatial distribution of the synthesized AgNPs. Most particles display a quasi-spherical geometry, while a limited fraction adopts slightly elongated or irregular polyhedral forms. Such morphological diversity is frequently encountered in biosynthesized silver nanoparticles.

Direct measurement from TEM images places the particle size predominantly within the range of ∼15–35 nm. The metallic cores are clearly resolved, allowing precise identification of individual nanoparticles.

A more rigorous statistical evaluation was carried out using ImageJ software based on multiple TEM images. The average particle diameter was determined to be 23.33 ± 6.44 nm, which aligns well with the visually observed size range. The relatively small standard deviation reflects a fairly tight distribution of particle sizes, with most particles clustered around the mean value.

At pH 7, the particle population remains relatively small and uniform. This condition favors rapid nucleation events, leading to the formation of numerous primary particles with limited subsequent growth.^45^ Similar size regimes have been reported in related systems, where controlled nucleation produces nanosized particles stabilized by organic species present in the extract.^46^

#### 3.2.2 Size distribution and colloidal evolution of AgNPs

##### 3.2.2.1 Size distribution

The size distribution derived from ImageJ analysis shows a concentrated population centered around ∼20–25 nm, consistent with the calculated mean diameter (23.33 nm). The histogram constructed from measured particles (Fig. 6B) exhibits a slightly asymmetric shape rather than a perfectly symmetric bell curve.

To better describe this distribution, both Gaussian and log-normal models were applied (Table S1, in SI). The log-normal function provides a closer fit to the experimental data, as reflected by a higher coefficient of determination (*R*^2^ = 0.9674), along with lower *χ*^2^ (3.8526) and AIC (800.64) values compared to the Gaussian model (*R*^2^ = 0.9335; *χ*^2^ = 6.0781; AIC = 802.43). This fitting behavior supports a log-normal distribution pattern, which is commonly associated with nanoparticle systems governed by stochastic nucleation and growth processes.

The asymmetric profile observed in Fig. 6B is characteristic of systems where particle growth proceeds at different rates after an initial nucleation stage. Most particles remain within a narrow size window, while a smaller fraction grows to larger dimensions, producing a right-skewed distribution. This interpretation is also consistent with the measured standard deviation (±6.44 nm), indicating slight polydispersity rather than a strictly monodisperse system.

Under more alkaline conditions (pH ≥ 8), the micrographs reveal an increased presence of clustered domains. Individual particles become less isolated, and the apparent size distribution broadens. Such evolution is consistent with classical nucleation–growth behavior reported for metallic nanoparticles.^47,48^

##### 3.2.2.2 Dispersion and aggregation

The nanoparticles are generally well separated across large regions of the grid. At the same time, distinct aggregation zones are visible. Within these areas, particles assemble into chain-like (“necklace-like”) arrangements or compact clusters.

This pattern reflects moderate colloidal stability. Attractive van der Waals interactions drive partial aggregation when repulsive forces are insufficient to fully stabilize the dispersion. Comparable structures have been reported in nanoparticle systems with limited surface stabilization.

Although the nanoparticles exhibited predominantly spherical morphology, moderate polydispersity and partial aggregation were still observed, particularly at prolonged reaction times and under highly alkaline synthesis conditions.

Aggregation becomes more pronounced as the pH increases. Larger clusters, reaching the submicron scale (∼300 nm), appear alongside heterogeneous assemblies. The shift can be linked to reduced electrostatic repulsion in alkaline media, which facilitates particle–particle interactions and eventual coalescence.^49^ Although phytochemical species act as capping agents, their stabilizing role weakens under strongly basic conditions, allowing aggregation to proceed more readily.^50^ Similar behavior has been documented across a range of AgNP systems under varying chemical environments.^46^

##### 3.2.2.3 Nucleation–growth evolution of AgNPs

The TEM observations provide insight into the structural evolution of the nanoparticles during synthesis. Two regimes emerge:

• At neutral pH (pH 7), nucleation dominates. Numerous small nuclei form rapidly, yielding a population of comparatively well-dispersed nanoparticles.

• At higher pH (≥9), growth and aggregation become more prominent. Particles interact, coalesce, and develop into larger, less uniform assemblies.

The coexistence of isolated primary particles and aggregated structures reflects a dynamic balance between nucleation, growth, and interparticle interactions. The log-normal size distribution obtained from ImageJ analysis further supports this interpretation, as such distributions typically arise from multiplicative growth processes rather than uniform particle formation. TEM characterization thus clarifies both the nanoscale dimensions and the statistical distribution of the AgNPs, while also capturing the transition from discrete particles to aggregated domains under changing chemical conditions.

#### 3.2.3 XRD patterns of the biosynthesized AgNPs

The XRD patterns of the biosynthesized AgNPs obtained at pH 7, 8, 9, and 10 are presented in Fig. 5e. Three well-defined diffraction peaks appear at 2*θ* values of 38.1°, 44.3°, and 64.4°, corresponding to the (111), (200), and (220) planes of face-centered cubic (fcc) silver (JCPDS card no. 04-0783).^51^ No additional reflections associated with silver oxides, such as Ag_2_O, are detected, indicating that the reduction process led predominantly to metallic Ag^0^. The plant extract maintained sufficient reducing strength throughout the synthesis, while simultaneously limiting oxidation under the reaction conditions.

As the pH increases, the diffraction peaks become progressively more intense and sharper. The reflections associated with the (200) and (220) planes exhibit noticeable narrowing at pH 9 and 10, pointing to enhanced crystallinity and growth of coherent crystalline domains. Such evolution in peak profile is typically associated with accelerated reduction kinetics under alkaline conditions, where both nucleation and subsequent growth proceed more rapidly.

Crystallite size was estimated using the Scherrer equation based on peak broadening analysis (Table S3 and Fig. S2, see in SI). The calculated sizes for the (111), (200), and (220) planes are 33.69, 27.19, and 17.76 nm, respectively, giving an average crystallite size of 26.21 ± 8.01 nm. Variations among planes reflect anisotropic broadening, which is commonly observed in nanoscale metallic systems.

A comparison with TEM-derived particle size (23.33 ± 6.44 nm) shows close agreement, with the XRD-based values slightly higher. This difference arises from the distinct measurement principles: TEM captures individual nanoparticles directly, while XRD evaluates coherent diffraction domains, which may include contributions from partially aggregated or internally structured regions. Despite this difference, both methods converge within the same nanoscale regime, supporting the formation of well-defined AgNPs.

Further structural insight is provided by the relative peak intensities and texture coefficient (TC) analysis (Table S4, in SI). The experimental intensity sequence deviates from the standard fcc pattern, with the (200) reflection exhibiting the highest intensity, followed by (220) and (111). Quantitative TC values reveal a preferred orientation along the (220) plane (TC > 1), whereas the (111) plane contributes less strongly compared to the standard reference. Although the (200) peak dominates in absolute intensity, its TC value remains lower than that of the (220) plane, indicating that texture evolution is governed by deviations from standard intensity ratios rather than peak magnitude alone.

Such preferential orientation suggests anisotropic crystal growth during nanoparticle formation. Differences in surface energy and growth kinetics among crystallographic planes likely drive this behavior, particularly under alkaline conditions where reaction rates are elevated.

The pH-dependent changes in peak intensity and narrowing further support enhanced crystallinity and structural evolution under alkaline synthesis conditions. These combined observations point to a transition toward larger, more heterogeneous nanoparticle populations as the synthesis environment becomes increasingly alkaline. The XRD results collectively confirm the formation of highly crystalline fcc AgNPs, with crystallite sizes consistent with TEM observations and evidence of pH-dependent structural evolution during nanoparticle growth.

#### 3.2.4 FTIR spectra of the biosynthesized AgNPs

The FTIR spectra of MCFE and the biosynthesized AgNPs shown in Fig. 5f exhibited multiple absorption bands within the range of 400–3600 cm^−1^, corresponding to various functional groups. The broad band observed between 3100 and 3400 cm^−1^ was assigned to O–H/N–H stretching vibrations associated with hydroxyl-containing phytochemicals. Noticeable differences in band shape, width, and position between MCFE and AgNP samples suggest interactions between these functional groups and the nanoparticle surface. The peak at 1401 cm^−1^ in MCFE corresponds to asymmetric CH_3_ bending or COO^−^ asymmetric stretching and became broadened into two adjacent peaks after AgNP formation. Several spectral features remained relatively unchanged, including the bands at 1644 and 1689 cm^−1^ attributed to C=C and C=O stretching vibrations, as well as the peaks at 2815 and 2980 cm^−1^ corresponding to aliphatic C–H stretching.

Additional spectral changes were observed after nanoparticle formation. The peaks at 1010 and 1087 cm^−1^, assigned to C–O–C and C–O–Ag stretching vibrations, exhibited substantial variations in relative intensity in the AgNP samples. Similarly, the aromatic C–H vibrations at 849 and 887 cm^−1^ decreased markedly in AgNPs8 and AgNPs9. Two low-wavenumber bands at 579 and 615 cm^−1^ were absent in MCFE but appeared in the AgNP spectra with varying intensity ratios. These bands are associated with X–Ag interactions (X = O or N), likely arising from coordination between Ag and oxygen-containing phenolic groups, generating weak bending vibrations in the low-frequency region.^52,53^ These spectral modifications support the involvement of phytochemical species as reducing and stabilizing agents during AgNP formation, with the most pronounced stabilization features observed for AgNPs7.

In comparison, the FTIR spectrum of chemically synthesized Ag obtained using NaBH_4_ displayed only a broad and symmetric absorption band centered around 3400 cm^−1^, most likely associated with adsorbed moisture, without clearly resolved features below ∼1000 cm^−1^ corresponding to Ag-related vibrations. The minimal spectral profile suggests the absence of stabilizing organic surface species in the chemically synthesized sample. Overall, the FTIR results indicate that while alkaline conditions (pH ≥ 9) promoted particle growth and aggregation, neutral conditions (pH 7) produced comparatively well-dispersed and stable AgNPs with smaller particle size. Therefore, AgNPs synthesized using 1.5 mL of 0.025 M AgNO_3_ and 2.5 mL of MCFE at pH 7 for 60 min (AgNPs7) were selected for further investigation due to their favorable combination of small size, narrow distribution, and high colloidal stability. Among the investigated conditions, AgNPs7 exhibited the most favorable combination of small particle size, narrow distribution, and colloidal stability, making this sample suitable for subsequent catalytic and physicochemical investigations.

### 3.3 Catalytic reduction

#### 3.3.1 Some factors affecting the catalytic reduction of RhB by AgNPs7

**Fig. 7:**
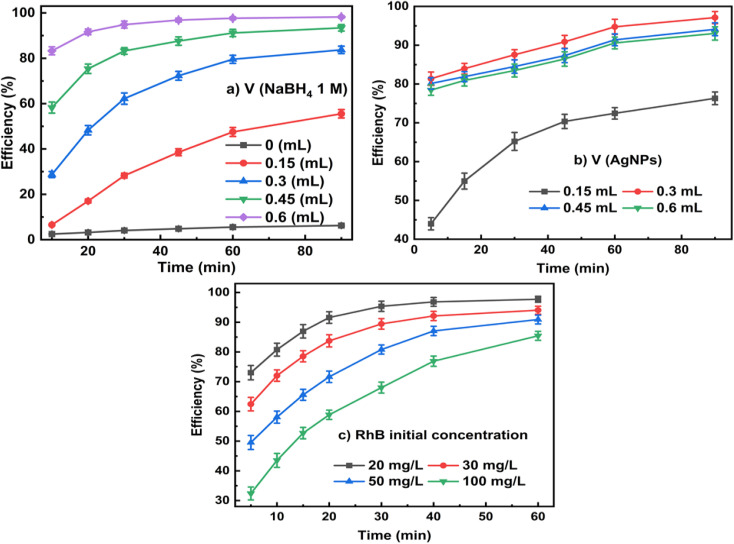
Effect of key reaction parameters on the catalytic reduction of Rhodamine B (RhB) in the presence of AgNPs-7: (a) volume of 1 M NaBH_4_, (b) volume of AgNPs-7 solution, and (c) initial RhB concentration. Data are presented as mean ± SD from three independent experiments (*n* = 3); error bars represent standard deviations.

The catalytic reduction performance of AgNPs7 toward Rhodamine B (RhB) was evaluated under different operational conditions, including NaBH_4_ dosage, catalyst loading, and initial RhB concentration. The corresponding degradation profiles are presented in Fig. 7.

##### 3.3.1.1 Effect of NaBH_4_ amount

Fig. 7a clearly indicates that RhB removal strongly depends on the amount of NaBH_4_. In the absence of NaBH_4_, negligible RhB removal (∼6%) was observed even after 90 min, indicating that AgNPs alone exhibited minimal catalytic activity under the investigated conditions. In the presence of 0.15 mL 1 M NaBH_4_ solution, the degradation process was still somewhat limited (about 21% at 30 min and about 55% at 60–90 min), indicating insufficient electron availability to efficiently sustain the reduction process. As the NaBH_4_ volume increased, the RhB removal efficiency was significantly improved. The best performance occurred at 0.60 mL NaBH_4_, removing more than 82% RhB after 10 minutes, 96.85% after 45 minutes, and slightly increased to 98.2% at 90 minutes. This behavior can be explained by the synergistic contributions of NaBH_4_ and AgNPs7.

##### 3.3.1.2 Effect of AgNPs7 amount

The impact of AgNPs7 dose on the catalytic degradation of 20 mg L^−1^ RhB in the presence of 0.6 mL NaBH_4_ is shown in Fig. 7b. As the volume of AgNPs7 was 1.5 mL, the RhB removal efficiency only achieved 44–76% over times of 5–90 min. When the catalyst dosage increased to 3 mL, the RhB removal efficiency was immediately achieved at about 82% within 5 min and near complete removal (98%) within 60 min, with near identical degradation following this time. However, further increasing the catalyst dosage to 4.5 or 6 mL resulted in decreased degradation performance, which plateaued at ∼89–90% after 60 min. This behavior is likely associated with nanoparticle aggregation at elevated AgNPs7 dosages and competition for available electrons supplied by NaBH_4_ under excessively catalyst-rich conditions. Thus, the optimal dosage of AgNPs7 catalyst is 3 mL, which will be selected for further studies.

##### 3.3.1.3 Effect of initial RhB concentration

Fig. 7c shows the effect of the initial RhB concentration on the catalytic reduction performance of AgNPs7 (3 mL) in the presence of 0.6 mL NaBH_4_. At an initial concentration of 20 mg L^−1^, the degradation occurred quickly – approximately 73% removal after the first 5 min and almost complete degradation (96.85%) after 40 min and then increased slightly when extended to 60 min (97.7%). When the initial RhB concentration increased, the efficiency decreased slightly. At an initial RhB concentration of 100 mg L^−1^, the degradation was limited to 32% at 5 min and 85.4% at 60 min, reflecting substrate overload relative to the available catalyst and electron donor. Nevertheless, the catalytic performance of the biosynthesized AgNPs7 remained superior to many previously reported green-synthesized AgNPs, both in terms of efficiency and reaction time: AgNPs were green synthesized using *Eucalyptus globulus* leaf extract, which was able to degrade up to 93% of 18 mg L^−1^ RhB at pH 12 after 80 min;^37^ AgNPs were green synthesized using *Matricaria chamomilla* L. leaf extract, which was able to degrade up to 93, 37% of 5 to 25 mg L^−^ RhB under UV irradiation after 130 min;^54^ AgNPs were green synthesized using *Eupatorium adenophorum* L. leaf extract, which was able to degrade up to 78.69% RhB under sunlight after 90 min;^55^ Similarly, AgNPs synthesized using Shorea robusta leaf extract achieved only 90.41% RhB degradation under comparable conditions.^56^

#### 3.3.2 Catalytic reduction kinetics

**Fig. 8:**
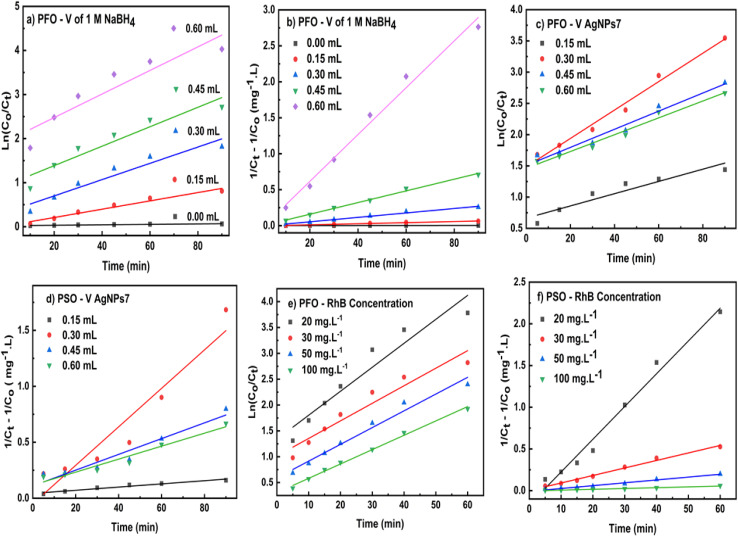
Kinetic modeling of Rhodamine B (RhB) catalytic reduction by AgNPs7 under different reaction conditions according to pseudo-first-order (PFO) and pseudo-second-order (PSO) models: effect of NaBH_4_ dosage fitted by (a) PFO and (b) PSO models; effect of AgNPs7 dosage fitted by (c) PFO and (d) PSO models; and effect of initial RhB concentration fitted by (e) PFO and (f) PSO models.

The catalytic reduction kinetics of RhB over AgNPs7 were evaluated using pseudo-first-order (PFO) and pseudo-second-order (PSO) kinetic models under different reaction conditions. The corresponding fitting profiles are presented in Fig. 8, while the calculated kinetic parameters are summarized in Table 2.

**Table 2:** Kinetic parameters of RhB catalytic reduction by AgNPs. Reported values are expressed as mean ± SD obtained from three independent measurements (*n* = 3)

		PFO kinetic model		PSO kinetic model	
		*K*_1_	*R* ^2^	*K*_2_	*R* ^2^
Volume of 1 M NaBH_4_ (mL)	0.00	4.921 × 10^−4^ ± 6.120 × 10^−5^	0.638	2.576 × 10^−5^ ± 3.133 × 10^−6^	0.944
	0.15	9.430 × 10^−3^ ± 8.403 × 10^−4^	0.969	7.573 × 10^−4^ ± 3.318 × 10^−5^	0.992
	0.30	0.0185 ± 0.0026	0.925	0.0031 ± 1.814 × 10^−4^	0.986
	0.45	0.0221 ± 0.0035	0.909	0.0081 ± 3.052 × 10^−4^	0.994
	0.6	0.0267 ± 0.0052	0.868	0.0342	0.987
Volume of AgNPs solution (mL)	1.5	0.0098 ± 0.0016	0.899	0.0014 ± 1.299 × 10^−4^	0.957
	3.0	0.0227 ± 0.0014	0.986	0.0172 ± 0.0027	0.911
	4.5	0.0145 ± 0.0012	0.972	0.0070 ± 9.354 × 10^−4^	0.934
	6.0	0.0135 ± 0.0010	0.978	0.0058 ± 6.536 × 10^−4^	0.952
RhB initial concentration (mg L^−1^)	10	0.0464 ± 0.0059	0.925	0.0393 ± 0.0023	0.983
	30	0.0340 ± 0.0041	0.931	0.0090 ± 3.708 × 10^−4^	0.992
	50	0.0324 ± 0.0023	0.975	0.0034 ± 1.857 × 10^−4^	0.985
	100	0.0270 ± 8.920 × 10^−4^	0.996	9.702 × 10^−4^ ± 7.040 × 10^−5^	0.974

Table 2 shows that: (1) as the amount of NaBH_4_ increased, the rate constant *k* increased steadily in both models → indicating that NaBH_4_ played an important role in providing electrons, which increased the catalytic efficiency. The *R*^2^ values in the PSO kinetic model are more than 0.98 showed that the PSO model described the experiment better. (2) As the solution increased from 1.5 → 3 mL, the rate constant increased sharply, then decreased as the volume of AgNPs7 volume of AgNPs7 continued to increase. *R*^2^ according to the PFO model is mostly higher than that in the PSO model; *R*^2^ according to the PSO model was in the range of 0.911 to 0.957. (3) With increasing RhB concentration, the rate constant decreases, which is usually due to the masking effect of the catalyst surface and the reduction of UV/vis light penetration. Both models give high *R*^2^ (>0.97), but PSO describes the experimental data more closely. Some studies have also obtained similar results.^57–59^

To facilitate more rigorous comparison of catalytic performance, the apparent pseudo-first-order rate constant was additionally considered relative to catalyst loading. Normalization of the kinetic response to catalyst amount suggests that the catalytic efficiency of the biosynthesized AgNP system remains competitive compared with previously reported phyto-mediated AgNP catalysts under comparable dye-reduction conditions. This behavior indicates that catalytic activity is influenced not only by the overall reduction rate, but also by nanoparticle accessibility, colloidal dispersion stability, and surface-mediated electron-transfer efficiency during the catalytic process.

However, the PSO model provided better agreement with the experimental data, suggesting strong contributions from adsorption-associated surface interactions and interfacial electron-transfer processes. Biosynthesized AgNPs are typically stabilized by phytochemical-derived organic surface species originating from the plant extract, which may influence both adsorption behavior and catalytic reduction kinetics. The improved agreement with the PSO model suggests that the catalytic reduction process is strongly influenced by adsorption-associated surface interactions and interfacial electron-transfer processes occurring at the AgNP interface. Similar PSO-dominated kinetic behavior has also been reported for other biosynthesized AgNP catalytic systems.^56,57^

Overall, the PSO model generally provided improved agreement with the experimental data, indicating that the catalytic reduction process is strongly influenced by adsorption-associated surface interactions and interfacial charge-transfer processes occurring at the AgNP interface.

#### 3.3.3 Mechanism of catalytic reduction

**Fig. 9:**
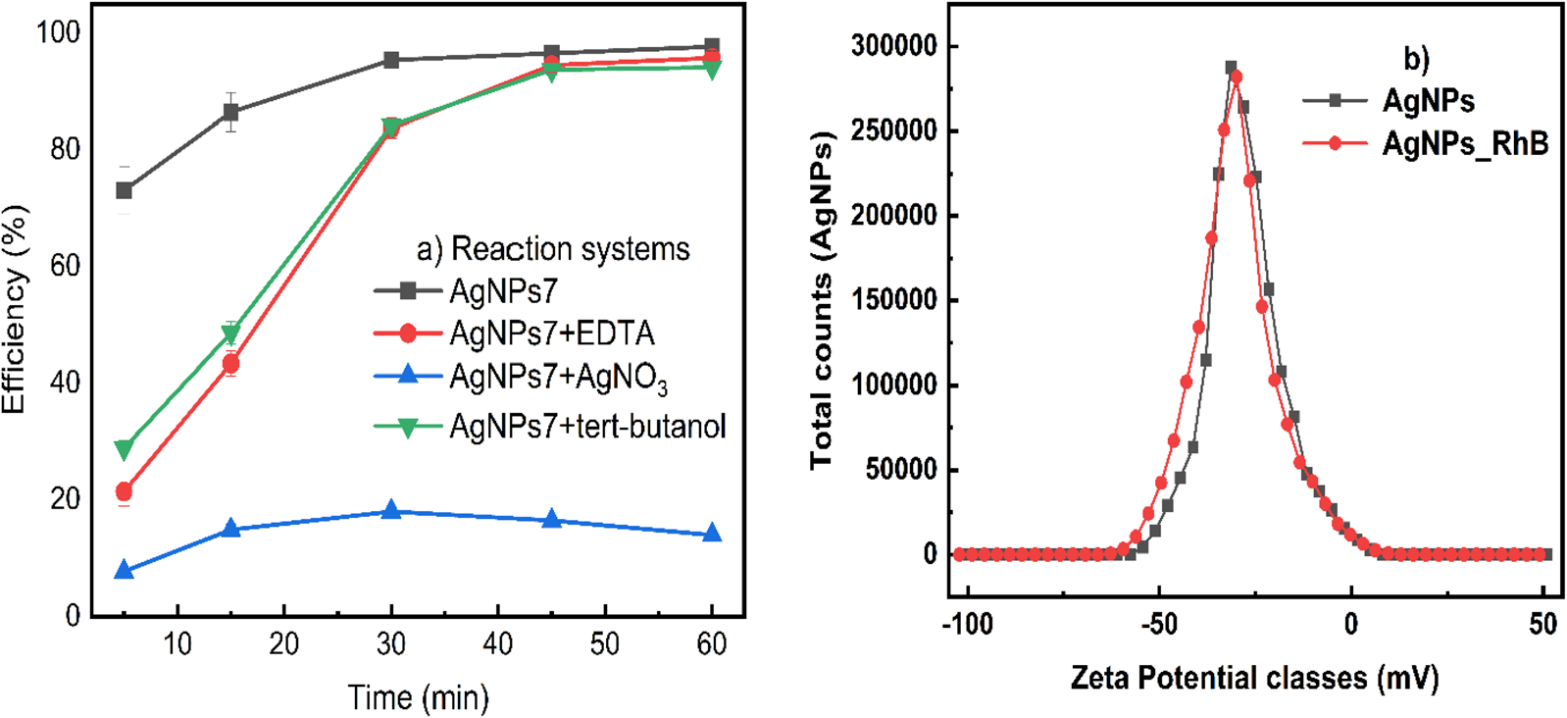
Effect of different scavengers on the catalytic reduction of RhB in aqueous solution by AgNPs7 (a) and zeta potential of AgNPs before and after removing RhB out of water (b). Data are shown as mean ± SD from three independent experiments (*n* = 3); error bars indicate standard deviations.

To further elucidate the catalytic reduction pathway of RhB over AgNPs7, mechanistic investigations were conducted using scavenger experiments and zeta potential analysis (Fig. 9). These analyses were combined with kinetic interpretation to better clarify the role of interfacial electron-transfer processes during catalytic reduction.

As shown in Fig. 9a, the absence of scavengers resulted in rapid RhB decolorization (∼98% within 60 min). The introduction of EDTA-2Na and *tert*-butanol induced a transient inhibition during the initial stage (20–30% removal at 5 min), followed by gradual recovery of catalytic efficiency to ∼94% at longer reaction times. In contrast, the presence of AgNO_3_, acting as a competing electron acceptor, drastically suppressed the reaction throughout the entire process (<20% efficiency). This preferential inhibition strongly suggests that interfacial electron transfer plays a dominant role in the catalytic process, whereas the contributions of holes and hydroxyl radicals are secondary and non-rate-determining.

The concentrations of EDTA-2Na, *tert*-butanol, and AgNO_3_ were chosen according to ranges commonly employed in mechanistic scavenger studies, while also considering the distinct inhibitory behavior associated with each compound. Since these scavengers interact with different reactive species and possess different scavenging efficiencies, their effects cannot be evaluated solely on the basis of absolute concentration. EDTA-2Na and *tert*-butanol primarily suppress hole- and hydroxyl-radical-associated pathways, respectively, leading mainly to temporary perturbation of secondary surface reactions during the early stage of RhB reduction. AgNO_3_ behaves differently. As a competing electron acceptor, it intercepts electrons transferred from BH_4_^−^ donor species across the AgNP interface. Persistent inhibition in the presence of AgNO_3_ is thus more consistent with disruption of the interfacial electron-transfer route governing RhB reduction than with a simple dosage-related scavenging effect. The catalytic reduction likely proceeds through an electron-relay mechanism consistent with a Langmuir–Hinshelwood-type surface process. In this mechanism, both BH_4_^−^ donor species and RhB molecules are co-adsorbed onto the AgNP surface, where AgNPs7 act as nanoscale electron-transfer mediators. Electrons supplied by BH_4_^−^ are transferred across the nanoparticle interface to RhB molecules, thereby facilitating reduction of the chromophoric structure into colorless products. Unlike conventional semiconductor photocatalysts such as TiO_2_ or ZnO, metallic AgNPs lack a bandgap due to overlap between the valence and conduction bands. Consequently, their catalytic behavior is governed primarily by interfacial electron-transfer processes occurring at the nanoparticle surface. Previous studies on plasmonic AgNP systems have proposed that localized surface plasmon resonance (LSPR) may participate in interfacial charge-transfer processes through transient high-energy electrons.^60,61^ Direct evidence for plasmon-assisted pathways was not obtained in the present work. Any possible plasmonic contribution is therefore discussed cautiously and only within the context of previously reported mechanistic interpretations.

Zeta potential analysis (Fig. 9b) shows negligible variation before and after the reaction, indicating that the interaction between RhB and the nanoparticle surface is weak and predominantly physisorptive. This observation, together with the negligible RhB removal (∼6%) in the absence of NaBH_4_, indicates that adsorption alone is insufficient and that the process is primarily governed by surface-mediated catalysis. Taken together, the results suggest that the catalytic process is dominated by interfacial electron-transfer interactions occurring at the AgNP surface, where AgNPs7 function as nanoscale electron mediators facilitating charge transfer between BH_4_^−^ donor species and RhB molecules. This behavior highlights a fundamental distinction between metallic nanoparticle catalysis and conventional semiconductor photocatalysis: rather than relying on band-structure-generated charge carriers, the reaction proceeds predominantly through localized interfacial electron-transfer processes occurring at the nanoparticle surface.

Under optimized synthesis conditions, improved colloidal stability and controlled nanoparticle growth may further enhance surface accessibility and interfacial charge-transfer efficiency.

**Fig. 10:**
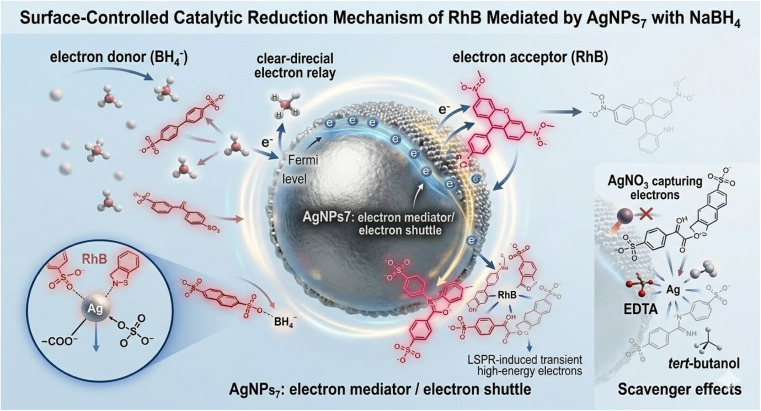
Proposed surface-mediated electron-transfer mechanism for the catalytic reduction of RhB by NaBH_4_ over biosynthesized AgNPs7, highlighting interfacial charge relay and scavenger-inhibited pathways.

Collectively, the scavenger experiments, kinetic interpretation, and zeta-potential analysis support a predominantly surface-mediated electron-transfer mechanism for RhB reduction over biosynthesized AgNPs7, as schematically illustrated in Fig. 10.

### 3.4 Antibacterial and cytotoxic performance

#### 3.4.1 Antibacterial activity

**Fig. 11:**
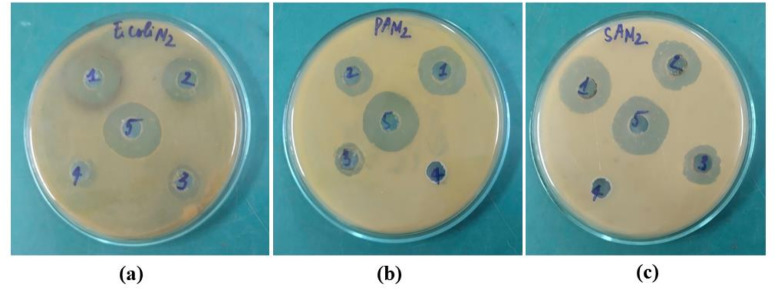
Antibacterial activity of AgNPs7 (1), AgNPs8 (2), MCFE (3), H_2_O (4) and antibiotic AMO (5) against *E. coli* (a), *PA* (b) and *SA* (c).

AgNPs8, with characteristics quite similar to AgNPs7 but slightly larger in size, was also evaluated for antibacterial activity (*E. coli*, *PA* and *SA*) along with AgNPs7, MCFE and antibiotic AMO by the agar disc diffusion method. Results are shown in Fig. 11 and Table 3.

**Table 3:** Inhibition zone diameter of test samples against *E. coli, S. aureus* and *P. aeruginosa.* Values are presented as mean ± SD from three independent experiments (*n* = 3)

No.	Samples	Concentration	Inhibition zone diameter (mm)		
			*SA*	*PA*	*E. coli*
1	AgNPs7	8 µg mL^−1^	20.3 ± 0.4	18.3 ± 0.4	21.7 ± 0.3
2	AgNPs8	8 µg mL^−1^	17.3 ± 0.4	14.0 ± 0.0	18.3 ± 0.4
3	MCFE	50 µg mL^−1^	11.3 ± 0.4	10.3 ± 0.4	12.7 ± 0.4
4	H_2_O	0	0.0 ± 0.0	0.0 ± 0.0	0.0 ± 0.0
5	AMO	100 µg mL^−1^	23.3 ± 0.4	23.0 ± 0.0	22.3 ± 0.6

Based on Table 3 and Fig. 11, both the silver nanoparticles and MCFE inhibited bacterial growth. MCFE alone (50 µg mL^−1^) produced only modest inhibition against all tested strains. Such behavior may arise from the limited dispersion stability and restricted membrane accessibility of freely dissolved phytochemicals in aqueous media. Once incorporated into the nanoparticle system, the antibacterial response increased markedly. AgNP formation likely altered the interfacial behavior of the phytochemical components, possibly improving nanoparticle dispersion and increasing interaction with bacterial surfaces. The phytochemical-derived organic corona may also contribute to colloidal stabilization and influence the local availability of Ag-active species at the nano–bio interface.

AgNPs8 displayed weaker antibacterial activity than AgNPs7 despite their comparable chemical composition. SEM observations together with UV-vis analysis revealed that AgNPs8 possessed larger particle sizes (∼30–50 nm), whereas AgNPs7 remained within a narrower nanoscale range (∼20–30 nm). Reduced antibacterial performance in AgNPs8 is plausibly linked to the lower surface-to-volume ratio and weaker interfacial interaction associated with larger nanoparticles. Smaller AgNPs generally provide a higher density of accessible surface sites and more intimate contact with bacterial envelopes. Better aqueous dispersion of AgNPs7 may also increase nanoparticle-cell encounters, reinforcing membrane-associated antibacterial interactions. The size-dependent behavior observed here aligns well with the established structure–activity relationship commonly reported for metallic nanomaterials.

The observed antibacterial activity likely depends not only on the intrinsic antimicrobial properties of silver, but also on nanoparticle size, colloidal stability, and surface-associated phytochemicals originating from the MCFE extract. Smaller and more uniformly dispersed AgNPs may provide higher surface accessibility and more effective interaction with bacterial membranes, which may favor membrane-associated interactions and oxidative-stress-related pathways. Residual phytochemical species adsorbed on the nanoparticle surface may additionally contribute to interfacial interactions with microbial cells and influence Ag^+^ release behavior.

AgNPs7 at 8 µg mL^−1^ generated inhibition zones comparable to those produced by amoxicillin (100 µg mL^−1^), reflecting broad-spectrum antibacterial behavior. Several mechanisms reported in previous studies may contribute to this enhancement, including electrostatic attraction between AgNPs and negatively charged bacterial membranes, gradual Ag^+^ release, localized membrane perturbation, and oxidative stress associated with reactive oxygen species (ROS) generation.^62,63^ No single pathway is likely to dominate completely. Instead, the antibacterial response probably emerges from coupled interactions occurring simultaneously at the nanoparticle surface. The phytochemical capping layer may further modulate nanoparticle dispersion and the accessibility of reactive Ag-associated surface sites. Direct mechanistic assays, including ROS quantification and membrane permeability analysis, were not performed in the present work. Accordingly, ROS generation, membrane disruption, and oxidative-stress responses should be regarded as literature-supported mechanistic hypotheses that are compatible with the observed antibacterial activity rather than experimentally verified mechanisms in the present study.

Antibacterial susceptibility followed the sequence: *S. aureus* > *E. coli* > *P. aeruginosa*. Similar trends have been described previously for AgNP systems.^64^ Structural differences in bacterial envelopes likely contribute to this variation. The porous peptidoglycan network of Gram-positive *S. aureus* may permit stronger interaction with AgNPs and Ag^+^ species. In contrast, Gram-negative bacteria possess an outer lipopolysaccharide membrane that partially restricts nanoparticle penetration. The comparatively lower susceptibility of *P. aeruginosa* may additionally relate to its strong biofilm-forming behavior, active efflux systems, and adaptive oxidative-stress defenses.

SEM observations (Fig. 5) suggested surface association between AgNPs7 and bacterial cells, accompanied by localized structural perturbation. Although ultrastructural damage was not quantitatively evaluated, the observed morphological alterations are consistent with antibacterial responses previously reported for AgNP systems; however, these observations alone cannot substantiate membrane disruption or ROS-mediated bacterial killing. Relative to several previously reported green-synthesized AgNP systems, AgNPs7 achieved comparable inhibition performance at substantially lower concentrations.^65,66^ Lower nanoparticle dosage is particularly relevant in biomedical and environmental contexts because it may reduce material consumption and limit unintended silver-associated toxicity. Molecular-level antibacterial pathways remain unresolved; nevertheless, the biosynthesized AgNPs displayed consistent broad-spectrum antibacterial activity closely linked to nanoparticle physicochemical characteristics.

#### 3.4.2 Cytotoxic activity on gastric cancer cell lines (AGS) and liver cancer cells (HepG2)

**Fig. 12:**
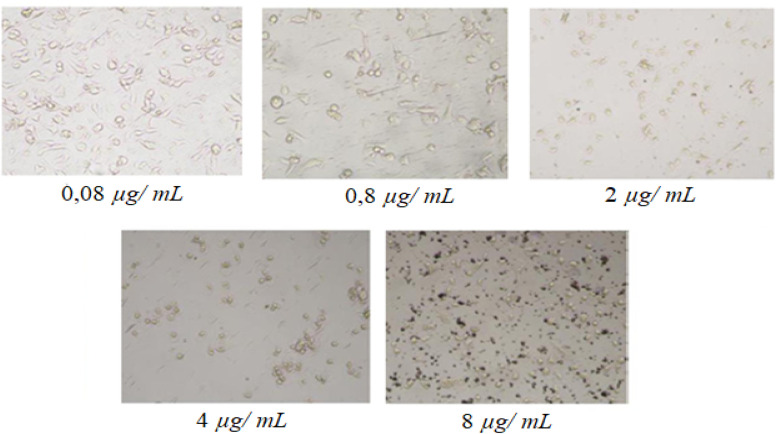
The cytotoxicity results of AgNPs7 on AGS cell line (magnification ratio 1 : 200).

**Fig. 13:**
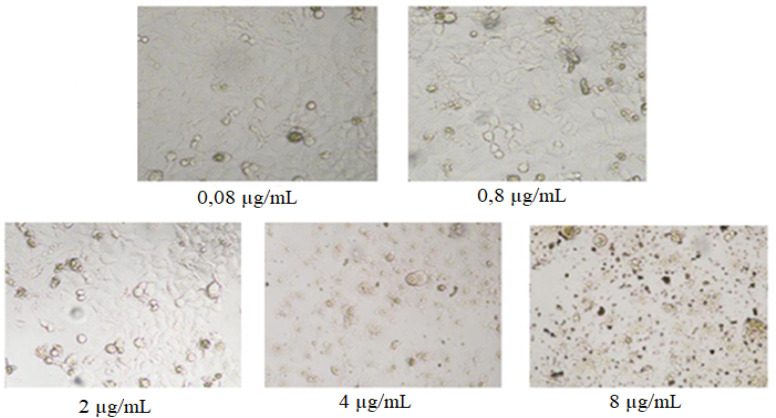
The cytotoxicity results of AgNPs7 on HepG2 cell line (magnification ratio 1 : 200).

The cytotoxicity of AgNPs7 on AGS and HepG2 cell line were evaluated by the MTT method. These results were also observed by microscopic examination in Fig. 12 and 13.

At low concentrations (0.08–0.8 µg mL^−1^), most HepG2 and AGS cells still retain their normal shape, with only scattered round cells appearing. When the concentration increases to 2–4 µg mL^−1^, cell density progressively decreases, and typical morphological signs of cell damage, such as shrinkage, rounding, and dark coloration became evident. The number of round cells that detach from the culture surface increases sharply. At a concentration of 8 µg mL^−1^, most of the cells were fully compromised, resulting in small, discrete fragments. This change in morphology supported the quantitative data and indicated the strong toxicity of AgNPs7 on cells that operated in a concentration-dependent fashion. These observations suggest that AgNPs exhibit significant cytotoxic activity and cytotoxic effects against AGS and HepG2 cells even at lower concentrations. The cytotoxic response may similarly be influenced by nanoparticle physicochemical properties, including particle size distribution, surface chemistry, and colloidal dispersion stability. AgNPs synthesized under optimized conditions exhibited comparatively controlled growth behavior and reduced aggregation, factors that may facilitate cellular interaction and nanoparticle internalization. In addition, phytochemical species associated with the nanoparticle surface may influence nano–bio interactions through modulation of oxidative stress pathways and interfacial cellular responses. Although the observed cytotoxic responses toward cancer cell lines are promising, evaluation using appropriate non-cancerous cell lines will be required before any conclusions regarding anticancer potential or tumor selectivity can be drawn.

**Fig. 14:**
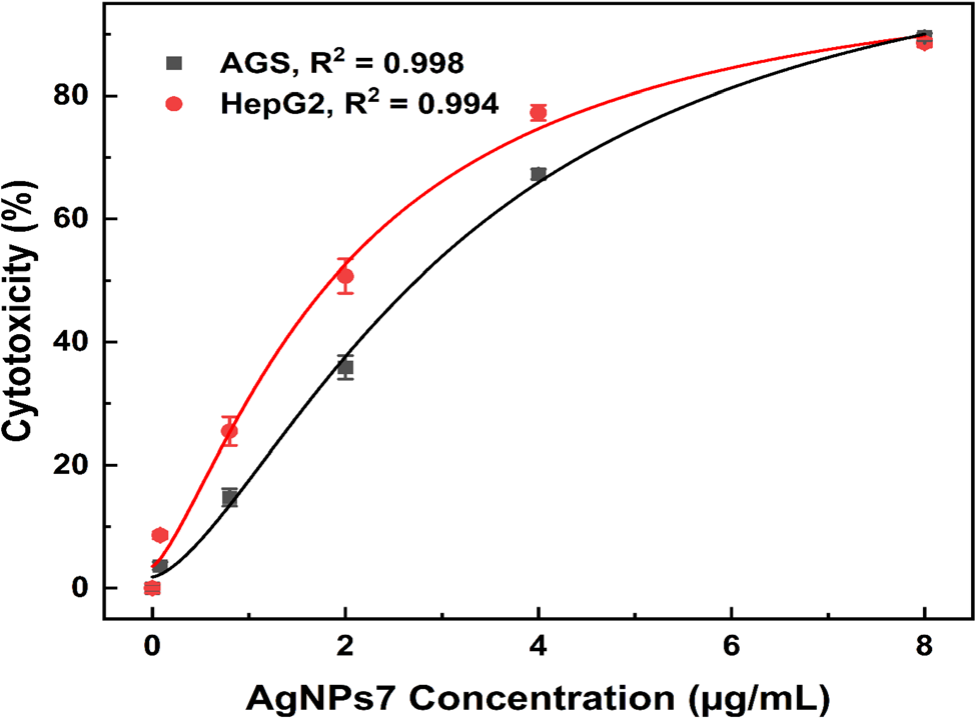
Cytotoxic effect of AgNPs7 on AGS and HepG2 cancer cells (fit according to 4PL model).

The data were fitted to the 4-parameter logistic model (4PL) according to the equation:10
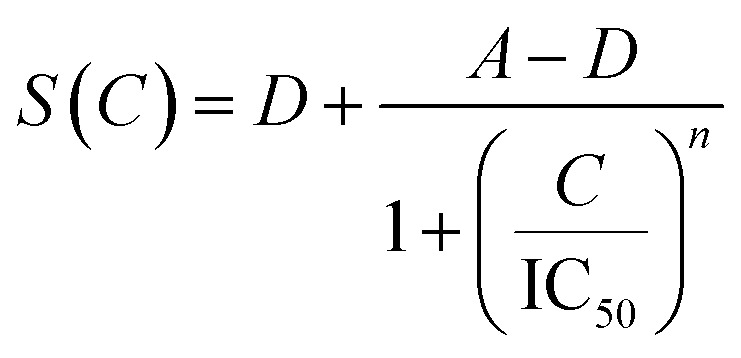
where *S*(*C*) is the proportion of viable cells at concentration *C*, *A* corresponds to the control sample (only the sample matrix) (100% viable cells); *D* is the response rate at the concentration (usually chosen = 0); IC_50_ is the concentration that causes a 50% reduction; *n* is the Hill coefficient – the slope of the curve, reflecting the mechanism of synergistic action.^67^ These results were shown in Fig. 14.

Fig. 14 showed that the experimental data fit well with the 4PL model. The cell viability decreased markedly with increasing AgNPs7 concentration for both AGS and HepG2 cell lines. The dose–response curves exhibited a sigmoidal trend, indicating a concentration-dependent cytotoxic effect. The Hill coefficients were 1.36 ± 0.38 for HepG2 cells and 1.53 ± 0.24 for AGS cells, suggesting a cooperative concentration-dependent cytotoxic response in which cell viability declined more rapidly after a critical concentration was reached rather than decreasing gradually. Similar threshold-like behaviour has previously been reported for AgNP systems and has been associated with mechanisms such as ROS generation, oxidative-stress accumulation, membrane damage, and subsequent cell death.^57^ However, these mechanisms remain literature-supported hypotheses and were not experimentally verified in the present study because intracellular ROS production, oxidative-stress biomarkers, and membrane integrity were not directly assessed. The HepG2 cells showed slightly higher sensitivity to AgNPs7, with cell death efficiency reaching nearly 90% at 8 µg mL^−1^, while AGS cells reached about 88% under the same conditions. The calculated IC_50_ value of approximately 2.05 ± 0.57 µg mL^−1^ (for HepG2 cell) and 3.22 ± 0.57 (AGS cell), suggests a potent cytotoxic activity of AgNPs7 toward both cancer cell types. The obtained IC_50_ values were comparatively lower than those reported for several previously published phyto-mediated AgNP systems, including AgNPs synthesized using aqueous extracts of *Pistacia atlantica* bark (IC_50_ 132 µg mL^−1^)^14^ or AgNPs were synthesised using extracts of *T. portulacastrum* and *C. Quinoa*.^68^

Compared with previously reported phyto-mediated AgNP systems, the biosynthesized AgNPs in the present study exhibited comparable or improved multifunctional performance under relatively mild synthesis conditions. The observed biological activity is therefore likely associated not only with silver-mediated effects, but also with the controlled colloidal properties and phytochemical environment generated during MCFE-mediated synthesis.

Papaya-mediated AgNP systems with comparable physicochemical characteristics have previously been reported to exhibit reduced IC_50_ values in cancer cell models relative to chemically synthesized AgNP counterparts.^69,70^ In one such system, papaya flower-derived AgNPs synthesized at pH 9 exhibited an average particle size of approximately 12 nm together with IC_50_ values of 21 µg mL^−1^ against MCF-7 cells and 169.96 ± 2.3 µg mL^−1^ toward HEK-293 cells.^70^ Direct comparison with the present study remains limited by differences in synthesis conditions, nanoparticle surface chemistry, and biological models. Nevertheless, these observations provide broader context for interpreting the cytotoxic responses observed in phyto-mediated AgNP systems.^71–73^

Previous studies have proposed that AgNP-associated oxidative stress, together with altered redox homeostasis and membrane damage, contributes to cytotoxicity in cancer cells. The present findings are compatible with these proposed mechanisms; however, intracellular ROS production, oxidative-stress signalling, and membrane damage were not directly examined and therefore remain literature-supported mechanistic hypotheses rather than experimentally verified pathways in this study. Such interfacial effects may contribute to differences in cellular susceptibility among distinct cell models. The low IC_50_ values observed for AGS and HepG2 cells are consistent with concentration-dependent *in vitro* cytotoxic responses under conditions commonly associated with nanoparticle internalization and subsequent cellular stress processes. Interpretation of these data nevertheless remains restricted to cancer cell models only. Evaluation using appropriate non-cancerous cell lines will be required for rigorous assessment of selectivity, biosafety, and biomedical relevance of the synthesized AgNPs.

### 3.5 Limitations and future perspectives

This study establishes a clear correlation between synthesis conditions, kinetic regimes and multifunctional performance of AgNPs; however, several key limitations remain.

The proposed nucleation–growth mechanism is inferred from *ex situ* spectroscopy, kinetic modelling and microscopy, and therefore lacks direct validation by *in situ* or *operando* techniques capable of resolving transient intermediates and dynamic growth pathways. In addition, the phytochemical composition of the extract was not quantitatively defined, limiting reproducibility and obscuring molecular-level structure–function relationships governing reduction and stabilization. In addition, phytochemical characterization of the extract remained limited to HPLC-DAD profiling and tentative assignment of several major flavonoid species, while comprehensive metabolite profiling and quantitative determination of individual phytochemical constituents were not systematically performed. This limitation restricts direct correlation between specific molecular components and the kinetic parameters governing nanoparticle nucleation, growth, and colloidal stabilization. From a catalytic perspective, the reliance on NaBH_4_ constrains scalability and decouples intrinsic plasmonic contributions from chemical reduction, thereby limiting mechanistic clarity. Catalyst durability, reusability and potential ion release were also not systematically assessed. In the present work, the biosynthesized AgNPs were intentionally employed as stable colloidal dispersions rather than recoverable heterogeneous catalysts. Due to the low nanoparticle loading and highly dispersed nature of the reaction system, quantitative recovery and reuse experiments could not be conducted reproducibly without perturbing nanoparticle concentration, dispersion stability, and surface chemistry. Furthermore, Ag^+^ release during catalytic and biological processes was not systematically quantified and therefore remains an important consideration for future environmental and biomedical assessment. Likewise, the long-term colloidal stability of the biosynthesized AgNPs under catalytic and biological conditions was not evaluated. Both Ag^+^ release and long-term colloidal stability may contribute to the observed catalytic efficiency as well as the antibacterial and cytotoxic responses and therefore warrant systematic investigation in future studies.

Furthermore, biological evaluations remain preliminary, as cytotoxicity was assessed only in cancer cell lines and no comparison with appropriate non-cancerous cells was performed. Consequently, the present findings do not establish tumor selectivity or justify conclusions regarding anticancer potential. Mechanistic investigations of ROS generation, membrane integrity, oxidative-stress signalling, and downstream cell-death pathways were beyond the scope of the present study. Consequently, the biological mechanisms discussed herein should be regarded as working hypotheses requiring dedicated mechanistic validation in future investigations. Quantitative antimicrobial evaluation also remains incomplete, as minimum inhibitory concentration (MIC) measurements were not systematically determined in the present study. In addition, the relationships between nanoparticle physicochemical properties, interfacial surface chemistry, and biological responses remain only partially resolved due to the complexity of nano–bio interactions under physiological environments. Finally, the absence of advanced surface analyses (*e.g.*, XPS) leaves the active surface chemistry insufficiently resolved.

Addressing these challenges requires a shift toward mechanistically informed and quantitatively controlled design. The integration of *in situ* diagnostics with predictive kinetic modelling represents a key challenge for resolving nucleation pathways in phyto-mediated systems. Quantitative phytochemical profiling and extract standardisation are required to enable reproducible and tunable synthesis. The development of self-sustained, light-driven catalytic systems that eliminate external reductants remains essential to decouple plasmonic effects and achieve sustainable operation. In parallel, rigorous evaluation of catalyst stability, performance in complex matrices (*e.g.*, real wastewater), and long-term behaviour is necessary to bridge laboratory findings with practical deployment. Future studies should therefore include systematic catalyst recycling experiments, ICP-based quantification of silver ion release, and long-term colloidal stability analyses under environmentally and biologically relevant conditions to clarify their respective contributions to catalytic and biological performance. Comprehensive biological evaluation should additionally incorporate MIC determination, comparative assessment using normal cell models, and systematic investigation of how nanoparticle size, colloidal stability, aggregation state, and surface-associated phytochemicals influence antibacterial and cytotoxic responses.

For biomedical applications, systematic interrogation of nano–bio interactions, toxicity pathways and therapeutic selectivity, including *in vivo* validation, is required.

Taken together, these directions will be critical to transform plant-mediated nanoparticle synthesis from an empirically driven process into a mechanistically predictable and application-oriented nanotechnology platform.

## 4 Conclusion

Ethanolic–aqueous extracts from male *Carica papaya* L. flowers provided a chemically active environment for controlled phyto-mediated synthesis of AgNPs under mild conditions. Systematic variation of synthesis parameters combined with time-resolved kinetic analysis showed that nanoparticle evolution extended beyond conventional endpoint-based descriptions commonly used in green synthesis systems.

Avrami exponents between 1 and 2 were consistent with discontinuous nucleation accompanied by low-dimensional, surface-influenced growth. Combined spectroscopic, microscopic, kinetic, and structural observations further support the interpretation that nanoparticle evolution involved coupled reduction–stabilization processes occurring at the growing AgNP interface. Under optimized conditions (pH 7, 2.5 mL extract, 1.5 mL AgNO_3_), the synthesized AgNPs exhibited crystalline face-centered cubic structures, narrow size distributions, and stable colloidal behavior.

The biosynthesized AgNPs showed rapid catalytic reduction of rhodamine B together with pronounced antibacterial activity against *Pseudomonas aeruginosa*, *Escherichia coli*, and *Staphylococcus aureus*, as well as measurable cytotoxicity toward AGS and HepG2 cells. The experimental observations are consistent with catalytic activity being dominated by surface-mediated electron transfer at the AgNP interface rather than conventional semiconductor-type photocatalytic pathways, with AgNPs acting as interfacial electron-transfer mediators in the NaBH_4_-assisted system.

Integrating kinetic descriptors with structural and functional characterization provides a more coherent structure–kinetics–function interpretation of phyto-mediated nanoparticle formation. Within this framework, nanoparticle growth and colloidal evolution can be interpreted in relation to controllable synthesis variables rather than empirical trial-and-error optimization alone.

Male *Carica papaya* flower extract also provided a chemically responsive reaction medium whose phytochemical composition appears to influence nanoscale growth and colloidal stabilization under the synthesis conditions examined. This observation further supports the emerging view of plant-derived extracts as chemically tunable environments for nanomaterial fabrication.

Further progress will benefit from establishing quantitative links among phytochemical composition, nucleation behavior, structural evolution, and functional performance through more comprehensive phytochemical characterization, *in situ* kinetic monitoring, and data-guided optimization. Such advances would strengthen mechanistic understanding while facilitating the rational development of environmentally compatible nanomaterials with tunable catalytic, antimicrobial, and biomedical properties.

## Author contributions

Thi Tam Khieu: methodology, data curation, formal analysis, validation, visualization, supervision, conceptualization, writing – original draft, writing – review and editing; Thi Hong Tham Diep: investigation, data curation, formal analysis; Ngoc Anh Hoang, Thi Thuy Ha Dinh: data curation, investigation; Thanh Hai Cao: investigation, data curation, visualization; Tuan Kien Vu: investigation, formal analysis, software; Quang Tung Vu: investigation; Le Phuong Hoang: validation, methodology, conceptualization; The Chinh Pham: methodology, project administration, resources; Truong Xuan Vuong: validation, visualization, writing – review and editing; Thi Thao Truong: supervision, validation, visualization, writing – review and editing.

## Conflicts of interest

The authors declare that they have no known competing financial interests or personal relationships that could have appeared to influence the work reported in this paper.

## Supplementary Material

RA-OLF-D6RA02406H-s001

## Data Availability

All data supporting the findings of this study are available within the article and its supplementary information (SI) (Tables S1–S4 and Fig. S1 and S2). Further information is available from the corresponding author upon reasonable request. Supplementary information: Table S1: HPLC-DAD chromatographic conditions and flavonoid retention times. Table S2: comparison of Gaussian and log-normal particle-size distribution models. Table S3: XRD-derived crystallite sizes of AgNPs. Table S4: texture coefficient (TC) analysis based on XRD data. Fig. S1: HPLC-DAD chromatogram of *Carica papaya* flower extract. Fig. S2: XRD pattern and crystallite-size analysis of the synthesized AgNPs. See DOI: https://doi.org/10.1039/d6ra02406h.
