# Supplementary material for: Green synthesis of silver nanoparticles using Carica papaya L. flower extract for catalytic reduction of rhodamine B and biological activities
Source: RSC Adv. 2026 Jul 15. Online ahead of print. doi: 10.1039/d6ra02406h (PMC13370743; doi:10.1039/d6ra02406h)
Supplement: RA-OLF-D6RA02406H-s001 [file RA-OLF-D6RA02406H-s001.pdf]

Supplementary Information (SI) for RSC Advances.
This journal is © The Royal Society of Chemistry 2026

SUPPLEMENTARY INFORMATION

Green Synthesis of Silver Nanoparticles Using *Carica papaya* L. Flower Extract for Catalytic Reduction of Rhodamine B and Biological Activities

Table S1. Chromatographic conditions of the HPLC-DAD analysis and retention times of quercitrin, quercetin, and kaempferol detected in papaya leaf extract.

Parameter / Compound	Condition / Retention Time
Analytical column	Reversed-phase C18 column (Zorbax Eclipse Plus C18, 4.6 × 250 mm, 5 µm)
Mobile phase	Solvent A: distilled water containing 0.1% formic acid; Solvent B: acetonitrile
Elution mode	Gradient elution
Flow rate	5.0 µL/min
Injection volume	10 µL
Detection wavelength (DAD)	254 nm
Quercitrin (RT, min)	17.439
Quercetin (RT, min)	20.718
Kaempferol (RT, min)	27.977

Table S2. Comparison of Gaussian and log-normal models for fitting particle size distribution of AgNPs.

Model	R^2	χ^2	AIC
Gaussian	0.9335	6.0781	802.43
Log-normal	0.9674	3.8526	800.64

The particle size distribution was fitted using both Gaussian and log-normal models. The log-normal model exhibited a better fit, as indicated by a higher coefficient of determination (R^2 = 0.9674), lower chi-square (χ^2 = 3.8526), and lower Akaike information criterion (AIC = 800.64), suggesting that the size distribution of AgNPs follows a log-normal behavior.

Table S3. Crystallite size of AgNPs calculated from XRD using the Scherrer equation

2θ (°)	FWHM (°)	Plane (hkl)	θ (rad)	β (rad)	Crystallite size, D (nm)
38.05	0.249	(111)	0.3321	0.00435	33.69
43.65	0.315	(200)	0.3809	0.00549	27.19
63.94	0.527	(220)	0.5580	0.00920	17.76
**Mean Crystallite size**					**26.21 ± 8.01**

Table S4. Texture coefficient (TC) analysis of AgNPs based on experimental and standard diffraction intensities.

(hkl)	I	I_0	I/I_0	TC
(111)	300	100	3.00	0.16
(200)	1000	46	21.74	1.19
(220)	600	20	30.00	1.64

The texture coefficient (TC) values were calculated based on the ratio of experimental intensities (I) to standard reference intensities (I_0) for different crystallographic planes.

Figures

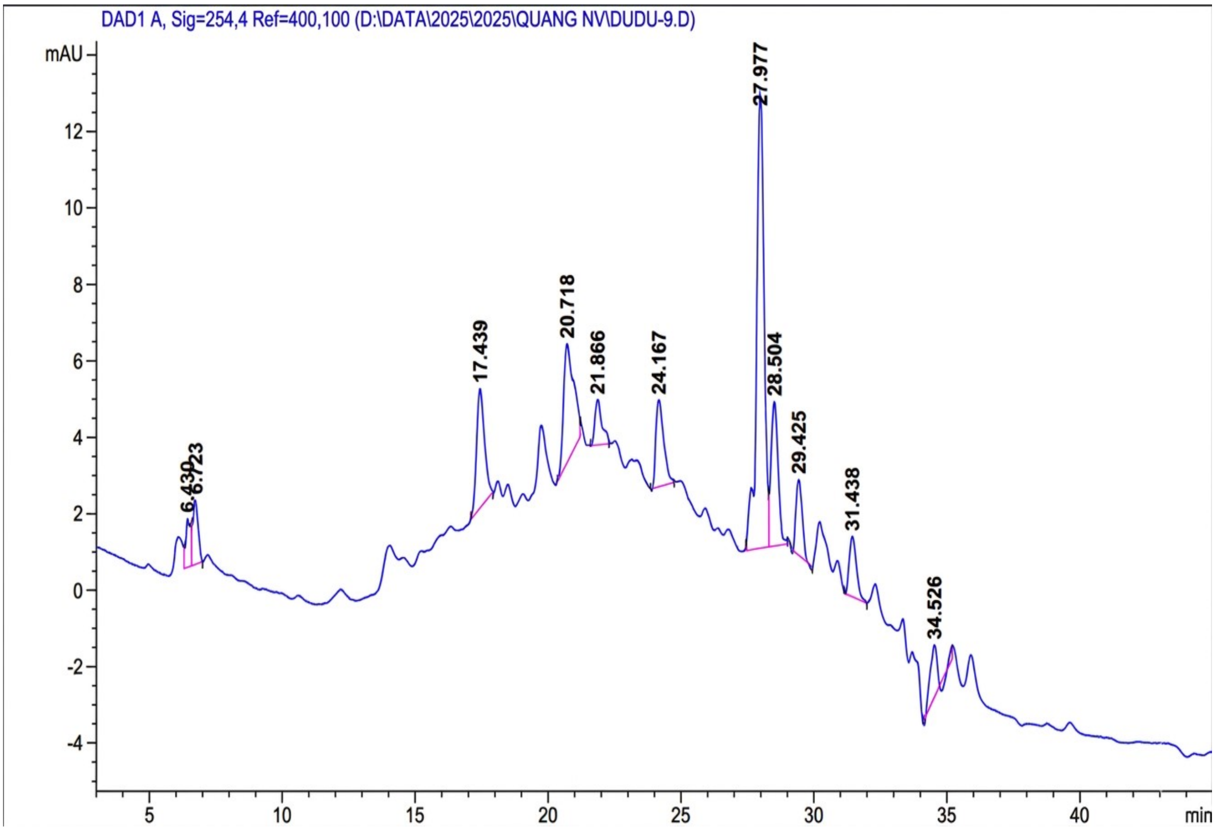

Fig. S1. HPLC-DAD chromatogram of flavonoid compounds detected in papaya leaf extract at 254 nm, showing the characteristic peaks of quercitrin, quercetin, and kaempferol.

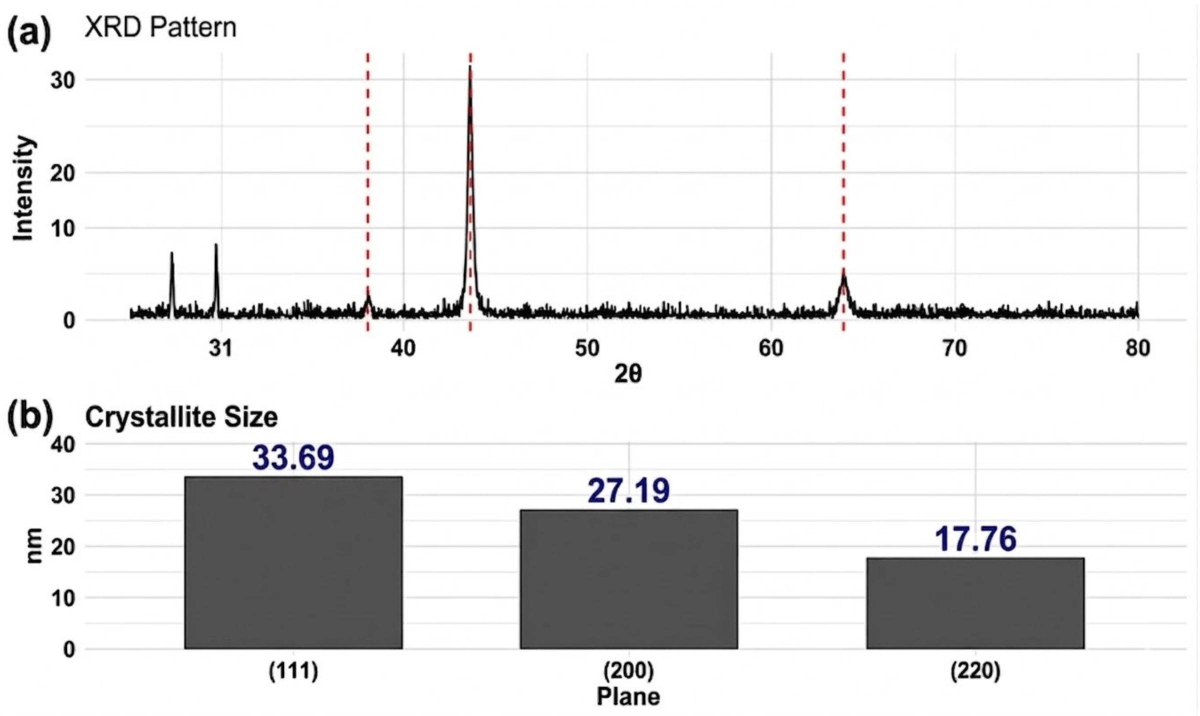

Figure S2. Characterization of the sample. **(a)** Measured X-ray Diffraction (XRD) pattern showing characteristic peaks at positions marked by red dashed vertical lines. **(b)** Summary of crystallite sizes (in nm) calculated from peak broadening for the (111), (200), and (220) crystallographic planes. Values are averages with no error bars shown for simplicity. Data labels in panel (b) indicate calculated values.
